# Stroke knowledge and intended care-seeking behavior among adults with hypertension and type 2 diabetes: A cross-sectional study

**DOI:** 10.1371/journal.pone.0335001

**Published:** 2026-10-27

**Authors:** Luisa Fernanda Alviz, Juan Diego Oyola, Juan Diego Martinez-Lemus, Javier Alexander Perez-Lopez, Mariana Medina-Soler, Jaime Eduardo Rodriguez, John Benavides, Carlos Martinez, Claudio Jimenez

**Affiliations:** 1 Department of Neurology, Columbia University Irving Medical Center/New York Presbyterian Hospital, New York, New York, United States of America; 2 Grupo de Investigación de Neuroepidemiología, Subred Integrada de Servicios de Salud del Norte E.S.E., Bogotá, Colombia; 3 Department of Neurology, Hospital Simón Bolívar, Subred Integrada de Servicios de Salud del Norte E.S.E, Bogotá, Colombia; 4 Department of Neurology, McGovern Medical School, UTHealth Houston, McGovern Medical School, Houston, Texas, United States of America; 5 Department of Clinical Neurological Sciences, London Health Sciences Centre, Western University, London, ON, Canada; 6 Departamento de Neurología, Fundación Santa Fe de Bogotá, Bogotá, Colombia; 7 School of Medicine, Universidad de los Andes Bogotá, Bogotá, Colombia; Southern Illinois University Carbondale, UNITED STATES MINOR OUTLYING ISLANDS

## Abstract

**Background:**

Stroke health literacy refers to the ability of non-medical individuals to recognize stroke symptoms and make timely decisions, which is critical for improving outcomes. However, data on stroke knowledge and intended care-seeking behavior in Bogotá, Colombia, remain limited.

**Objective:**

To assess stroke symptom knowledge and intended care-seeking behavior among adults with hypertension and type 2 diabetes and to identify sociodemographic predictors of limited knowledge and suboptimal response.

**Methods:**

A cross-sectional study was conducted between October 2023 and April 2024 in Bogotá, Colombia. A structured questionnaire was administered to 5,402 adults with hypertension and/or type 2 diabetes. Participants were recruited using a non-probabilistic sampling strategy, primarily from individuals under routine follow-up within the Bogotá public health network, and were identified through multiple recruitment strategies, including institutional databases. Surveys were conducted either by telephone or in person. Continuous variables were summarized using descriptive statistics, differences in categorical variables were assessed using chi-square tests, and binomial logistic regression models were applied to evaluate adjusted associations.

**Results:**

Of 6,050 respondents, 5,402 met inclusion criteria (median age 67 years; 46.3% male). Although most participants recognized that ischemic stroke can cause disability (89.6%) and death (91.1%), 32.5% reported no knowledge of stroke symptoms. Limited symptom knowledge was independently associated with male sex (OR 1.26, 95% CI 1.12–1.42), low educational attainment (OR 1.59, 95% CI 1.38–1.82), and low socioeconomic status (OR 2.20, 95% CI 1.85–2.60). Regarding intended response, 26.6% would not activate emergency medical services, and 52% reported they would schedule an outpatient appointment instead. Lower educational attainment was associated with greater likelihood of reporting non-emergency responses (all p < 0.001).

**Conclusions:**

Despite high awareness that stroke can cause disability and death, substantial gaps in symptom recognition and intended response persist, particularly among socioeconomically disadvantaged individuals. These findings highlight the need for targeted strategies to improve stroke literacy in high-risk populations.

## Introduction

According to the World Health Organization (WHO), approximately 15 million individuals experience an ischemic stroke (IS) each year, resulting in nearly 6 million deaths and leaving an additional 5 million individuals with disability [1]. Globally, IS accounts for an estimated 160.4 million disability-adjusted life years (DALYs), ranking it among the leading causes of mortality and disability worldwide [2]. These figures highlight the substantial global burden of stroke, and underscore the importance of early diagnosis and timely treatment.

The burden of IS is particularly relevant in lower- and middle-income countries (LMICs), where disparities in healthcare and limited implementation of effective prevention strategies may increase the risk of adverse outcomes. Although IS is a time-sensitive condition that can be effectively treated within the first hours of symptom onset, and is largely preventable through population-level interventions targeting modifiable risk factors, the implementation of such strategies remains uneven across regions [3].

This variability may be partly attributable to gaps in understanding the stroke care continuum in these regions, due to limited research evaluating gaps that occur from the presence of risk factors and symptom recognition, to timely healthcare access and treatment. Identifying and characterizing these gaps is essential to inform targeted interventions aimed at mitigating stroke burden.

In Colombia, the absolute number of IS cases has increased, driven by population growth, increased life expectancy, and improved detection within medical services [4]. However, it remains unclear whether reductions in mortality have translated into meaningful decreases in IS-related disability, underscoring the need to strengthen prevention and early response strategies.

A critical component influencing outcomes across the stroke care continuum is health literacy, which encompasses an individual’s understanding of the disease, recognition of warning symptoms, and the ability to promptly seek medical care [5]. International studies consistently demonstrate substantial gaps in stroke awareness. For instance, a study conducted in Dhulikhel, Nepal, reported that 60% of participants were unaware of stroke risk factors, only 16% recognized sudden unilateral weakness as a warning sign, and 64% were unaware of available stroke treatments (n = 273) [6]. Similarly, a study conducted in Al-Ahsa, Saudi Arabia, found that 56.9% of participants lacked knowledge regarding stroke risk factors, warning signs, and prevention strategies (n = 202). Higher levels of awareness were observed among older adults, individuals with higher socioeconomic and educational backgrounds, and those with a family history of IS, suggesting that health literacy is related to social determinants of health [7].

In Colombia, stroke awareness remains limited. A study conducted in Manizales, a mid-sized city in the Andean region, found that only 11.7% of participants reported they would seek emergency care if experiencing symptoms of an acute IS [8]. Despite these findings, evidence remains scarce in Bogotá, particularly among individuals with established vascular risk factors.

To address this gap, the present study aims to identify factors associated with stroke-related knowledge and intended emergency care-seeking behaviors among patients diagnosed with type 2 DM or systemic arterial HTN receiving care within the public healthcare network of Bogotá, Colombia. By focusing on individuals at elevated vascular risk, this study seeks to generate context-specific evidence to inform targeted public health strategies aimed at strengthening stroke prevention and improving timely recognition and response.

## Methodology

### Definitions and concepts

In Colombia, socioeconomic status is determined through a six-level residential stratification system (“estratos”; singular: estrato) based on household income, housing characteristics, and neighborhood infrastructure [9]. This system directly informs the allocation of public service subsidies and user co-payments, including healthcare, transportation, and utilities. Stratum 1 corresponds to the lowest socioeconomic level and Stratum 6 to the highest. The model is intended to promote equity by redistributing costs, whereby higher strata assume proportionally greater financial contributions and lower strata receive targeted subsidies [10].

The Colombian education system is structured into two main levels: basic and higher education. Basic education comprises five years of primary school, followed by six years of secondary education. Higher education is delivered through universities and technical institutions and encompasses undergraduate, professional, and postgraduate training, with doctoral degrees representing the highest academic qualification [11].

Healthcare delivery in Bogotá is organized through Health Service Provider Institutions (Instituciones Prestadoras de Salud, IPS), which operate under the public health network or the private sector. The public network is divided into four geographic subnetworks: north, south, southwest, and center-east (Fig 1). As of 2016, Bogotá had 22 hospitals and 142 health centers. Between 2020 and 2024, seven additional hospitals and 20 health centers were incorporated into the network [11,12].

**Fig 1:**
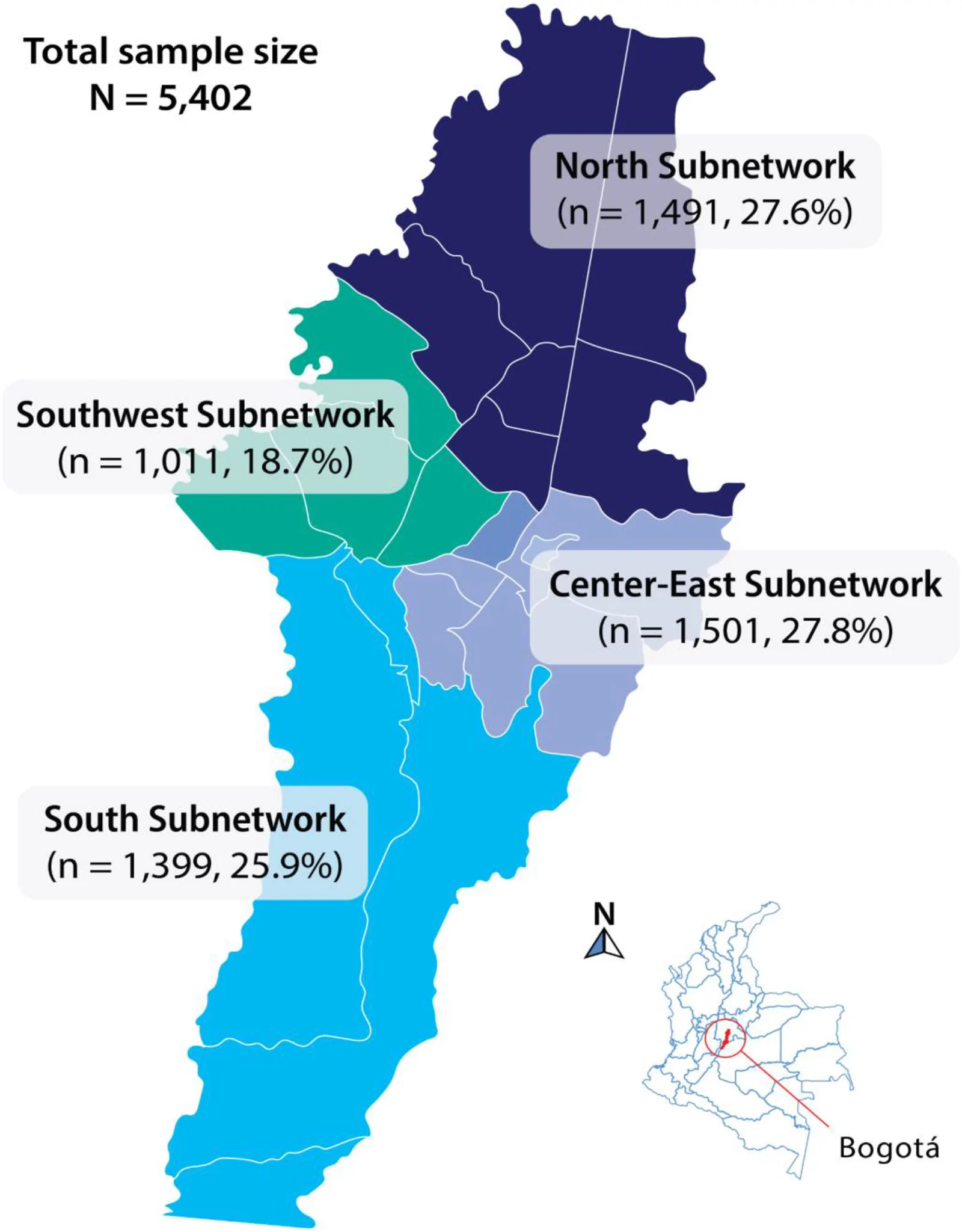
Geographic distribution of the four Integrated Health Subnetworks in Bogotá. This Fig presents a map of Bogotá illustrating the distribution of the four main health service subnetworks. Among the 5,402 included participants, 27.6% lived in areas covered by the North Subnetwork, 18.7% by the Southwest Subnetwork, 27.8% by the Center-East Subnetwork, and 25.9% by the South Subnetwork. Originally designed by Freepik – Magnific.com [14]; modified by the authors.

Management of chronic non-communicable diseases, including hypertension and type 2 diabetes mellitus, is guided by national Clinical Practice Guidelines (Guías de Práctica Clínica, GPC) issued by the Ministerio de Salud y Protección Social. These guidelines structure outpatient follow-up, risk stratification, and longitudinal cardiovascular risk management, primarily within primary care–based programs supported by integrated electronic medical records [13]. No publicly available data specify how many facilities are equipped to provide acute reperfusion therapies for IS, including intravenous thrombolysis or mechanical thrombectomy.

### Study design, population, and eligibility criteria

An analytical cross-sectional study was conducted in Bogotá, Colombia, between October 2023 and April 2024. Participants were recruited from University Hospital Fundación Santa Fe de Bogotá (UHFSFB) and hospitals within the governmental Integrated Health Subnetworks of the North, South, and Center-East (Fig 1), as well as through complementary community-based strategies.

#### Eligibility criteria.

Eligible participants were adults aged ≥18 years with a documented diagnosis of type 2 DM or HTN who had resided in Bogotá for at least one year. These conditions were selected because they are major modifiable risk factors for stroke and are managed through structured outpatient chronic disease programs aligned with national clinical practice guidelines for non-communicable diseases [13]. In Bogotá, patients with HTN and DM are routinely followed in primary care–based programs supported by an integrated electronic medical record system, which enables systematic identification and recruitment of individuals at elevated vascular risk.

#### Exclusion criteria.

Patients with any prior history of stroke, cerebrovascular event, or any disease that compromises the mental sphere, such as dementia or any degree of intellectual disability.

### Sample size determination

A stratified non-probability sampling approach by community areas was used. Sample size estimation was based on projected hypertension prevalence in Bogotá (24%), given the absence of equally robust population-level estimates for diabetes prevalence [15]. Because both HTN and type 2 DM are strongly associated with IS risk and define the target high-risk population, HTN prevalence was used as a pragmatic proxy for estimating the required sample size. Using population projections from SaluData, the estimated number of adults with hypertension in Bogotá was 1,442,929 [15]. Under these assumptions, a minimum sample size of 5,402 participants was calculated to achieve a 95% confidence level with an estimated margin of error of 1.18%. This precision should be interpreted as an approximate justification of sample adequacy rather than a strict inferential guarantee, particularly given the inclusion of participants with either hypertension or diabetes and the non-probability nature of the sampling design.

Because participants were not randomly selected, the sample may not be fully representative of the broader population of individuals with these conditions. Accordingly, findings should be interpreted with consideration of potential selection bias and limited external validity.

### Development and validation of the data collection instrument

The questionnaire was developed through a structured, multi-step process. First, a comprehensive literature review was conducted to identify key domains and previously used measures related to stroke knowledge, attitudes, and practices. Relevant articles were identified using Medical Subject Headings (MeSH) and Descritores en Ciencias de la Salud (DeCS) terms, combining concepts related to chronic diseases, stroke, and health literacy. The reviewed literature informed the conceptual framework and item generation for the instrument.

The draft questionnaire was subsequently evaluated for content validity by a panel of neurologists and epidemiologists, who assessed clarity, relevance, and comprehensiveness. Seven pilot tests were conducted with graduate students in Epidemiology to refine wording, structure, and response options prior to implementation. Questions regarding risk factors for stroke were structured as dichotomous (‘yes’ or ‘no’) items. In contrast, questions assessing knowledge and intended care‑seeking behavior related to IS were graded on a five‑point Likert scale, varying between ‘totally agree’ and ‘totally disagree’ within a given statement.

Internal consistency was assessed using Cronbach’s alpha. The knowledge domain related to acute stroke demonstrated high reliability (α = 0.90). In contrast, the practices domain showed low internal consistency (α = 0.30), indicating limited reliability. Accordingly, the instrument should be considered partially validated, and findings derived from the practices domain should be interpreted with caution.

### Data collection

Data were collected between October 2023 and April 2024 by trained registered nurses affiliated with the participating institutions. Nurses received standardized instruction on questionnaire administration prior to study initiation.

#### Institutional recruitment.

Patients registered in chronic non-communicable disease databases at UHFSFB and the North, South, and Center-East health subnetworks were screened to identify individuals with a documented diagnosis of type 2 DM and/or HTN who were actively attending follow-up appointments in primary care, internal medicine, or general medicine clinics. The databases were filtered to obtain the most up-to-date contact information. Eligible patients were contacted by telephone and invited to participate. In addition, on-site recruitment was conducted among eligible patients attending participating hospitals, including outpatient clinics, inpatient services, emergency department waiting areas, and dialysis units.

#### Community-based recruitment.

To ensure inclusion of individuals not captured in institutional registries, complementary recruitment strategies were implemented in community settings, including churches, academic events, and public spaces. Individuals meeting inclusion criteria were invited to voluntarily complete the survey.

#### Survey administration and data management.

After explanation of the study objectives, confidentiality measures, and voluntary nature of participation, informed consent was obtained from all participants prior to questionnaire administration. Surveys were conducted either by telephone or in person. Responses were recorded electronically using Google Forms, facilitating secure data storage and centralized management. At the conclusion of each survey, nurses delivered a brief standardized educational message reinforcing recognition of stroke warning symptoms, the importance of managing modifiable risk factors (HTN, DM, smoking, obesity), and the need to seek urgent medical care if symptoms occur.

#### Quality control.

Weekly monitoring meetings were conducted to review data completeness and questionnaire volume. Periodic random audits were performed by re-contacting selected participants to verify accurate questionnaire administration. Identified inconsistencies were reviewed and corrected to maintain data integrity and quality standards.

### Data analysis

Categorical variables (nominal and ordinal) were summarized as proportions. Continuous variables were assessed for normality using the Kolmogorov-Smirnov test and are presented as medians with interquartile ranges, as none followed a normal distribution.

For analytical purposes, selected sociodemographic variables were dichotomized. Low socioeconomic status was defined as residential stratum ≤2; low educational level as elementary school or less; and low income as ≤1,000,000 Colombian pesos per month. Although the residential stratification system may not perfectly reflect individual wealth in all settings [16,17], analyses were conducted separately by socioeconomic stratum and income to account for potential variability in social classification. Dichotomization was performed for analytical simplicity, acknowledging that this approach may reduce precision and obscure potential gradients in stroke knowledge and practices. Likewise, responses to the Likert‑scale items on intended care‑seeking behavior were dichotomized, with ‘totally agree’ coded as 1 and all other responses coded as 0. This decision reflected the observed hesitation among participants to select options other than ‘totally agree,’ and was intended to provide a practical and efficient summary of overall care‑seeking intentions in our sample. Nonetheless, we recognize that such coding may introduce bias and attenuate the richness of the original scale.

Bivariate comparisons were performed using a chi-square test. Statistical significance was defined as a p-value < 0.05. Statistical analysis was performed with SPSS software, version 25.

Associations between explanatory variables and stroke knowledge were estimated using binomial logistic regression. Odds ratios (ORs) and 95% confidence intervals (CIs) were obtained by exponentiating regression coefficients. Models were adjusted for age, sex, and diagnosis of HTN. Both crude and adjusted estimates are presented. Model fit was assessed using the Hosmer-Lemeshow goodness-of-fit test.

### Ethical considerations

Ethical approval was obtained from the institutional review boards of UHFSFB and the participating hospitals within the governmental health subnetworks of the North, South, and Center-East (Serial number: SNACEI-132. File 73). Written informed consent was obtained from all participants prior to questionnaire administration. For participants interviewed by telephone, verbal informed consent was obtained prior to data collection in accordance with the protocol approved by the ethics committee. Confidentiality was maintained throughout data collection, storage, and analysis in accordance with institutional guidelines.

### Inclusivity in global research

Additional information regarding the ethical, cultural, and scientific considerations specific to inclusivity in global research is included in the Supporting Information (S1 Checklist)

## Results

A total of 6,050 individuals with HTN and/or type 2 DM were recruited across the North, South, and Center-East Integrated Health Subnetworks of Bogotá and completed the questionnaire. Of these, 5,402 met inclusion criteria.

The median age was 67 years (IQR 59.68–74.94), and 46.3% (2502/5402) were male. One patient self-identified as intersex, and 1 patient chose to not disclose their sex. Due to the very low frequency of these categories, both were excluded from inferential analyses but retained in the descriptive statistics to ensure that the diversity of responses was represented. Median Body Mass Index (BMI) was 26.9 (IQR, 24.65–29.30). Most individuals belonged to low socioeconomic strata (81.6%, 4406/5402) and were enrolled in the subsidized health insurance regimen (92.4%, 4992/5402). More than half (55.3%, 2988/5402) had completed only elementary education. Detailed sociodemographic characteristics are presented in Supplementary Table S2 in S1 File.

Hypertension was present in 92.2% of participants, with no significant differences by sex (91.6% vs 92.7% in men and women, respectively, p = 0.154). Type 2 DM was reported in 32.1% of cases and was more frequent among men than women (34.0% vs 30.5%, p = 0.007). Self- reported cholesterol or triglyceride abnormalities (any dyslipidemia) were noted in 39.4% of participants and were more frequently reported by women than men (44.5% vs. 33.5%, p < 0.001). Current smoking was more common among men (5.9%) compared with women (2.2%)(p < 0.001) [Table 1].

**Table 1:** Questions about self-reported comorbidities.

Comorbidities	Women n/n (%)	Men n/n (%)	Total n/n (%)	*p*-value*
**Hypertension**	2686/2898 (92.7)	2292/2502 (91.6)	4980/5402 (92.2)	0.141
**Diabetes**	885/2898 (30.5)	849/2502 (34.0)	1734/5402 (32.1)	0.008
**Any Dyslipidemia**	1288/2897 (44.5)	839/2501 (33.5)	2129/5400 (39.4)	< 0.001
**Cigarette smoking**	65/2898 (2.2)	147/2502 (5.9)	212/5402 (3.9)	< 0.001

^*^Corresponding statistical test, significant at p < 0.05. One male participant reported no knowledge of dyslipidemia (n = 2,501) and one female participant declined to answer (n = 2,897).

### Knowledge of ischaemic stroke

Among responders with complete data, 89.6% (4837/5398) totally agreed that IS can lead to disability, and 91.1% (4917/5399) totally agreed that it may result in death. However, 32.6% (1764/5402) reported being unaware of any symptoms of IS. Reporting no knowledge of any stroke symptom was more frequent among men than women (36.5% [913/2502] vs. 29.1% [850/2898], p < 0.001). Multivariable logistic regression analysis reporting no knowledge of any IS symptom was significantly associated with male sex, low educational attainment, and lower socioeconomic status (Fig 2).

**Fig 2:**
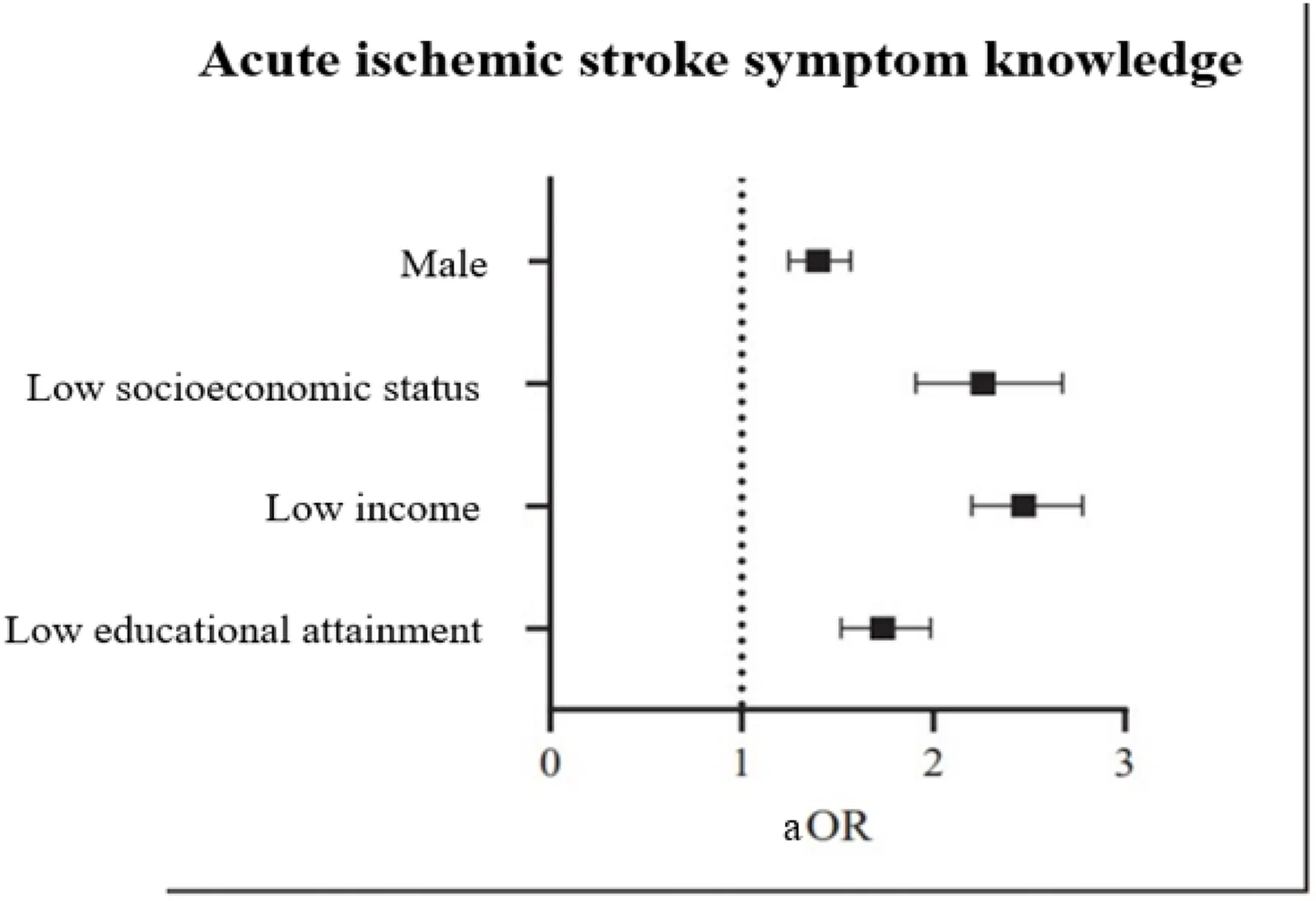
Predictors of Knowledge of Ischemic Stroke Symptoms. Summary of estimated association between acute ischemic stroke symptom knowledge among participants and sociodemographic variables. The odds Ratio (aOR) was adjusted for age and past medical history of systemic arterial hypertension.

### Intended care-seeking responses to Suspected Stroke

Regarding intended care-seeking responses to a suspected stroke, 26.6% (1446/5400) of participants did not express complete agreement (’totally agree’) with activating the emergency medical system by calling the emergency hotline. Men were more likely than women to express complete agreement with calling the emergency services hotline (p = 0.004). Despite this, 48% (2590/5400) of all respondents totally agreed that they would schedule a medical appointment if they suspected having an IS. This preference was significantly more common among men (p < 0.001) [Table 2].

**Table 2:** Intended care-seeking responses regarding emergency care for stroke.

Practice	Women n/n (%)	Men n/n (%)	Total n/n (%)	*p*-value*
**Call emergency hotline**	2075/2897 (71.6)	1880/2501 (75.2)	3956/5400 (73.3)	0.004
**Make a medical appointment**	1177/2897 (40.6)	1711/2501 (56.4)	2590/5400 (48)	< 0.001
**Go to the pharmacy**	510/2896 (17.5)	621/2501 (24.8)	1131/5399 (20.9)	< 0.001
**Take a home remedy**	518/2897 (17.8)	488/2501 (19.5)	1006/5400 (18.6)	0.116

*Corresponding statistical test, significant at p < 0.05. Two participants (one male, one female) declined to respond to items regarding emergency hotline activation, scheduling a medical appointment, or using home remedies, while three participants (two females, one male) declined to respond to the item regarding pharmacy visits.

In adjusted analyses, non-emergency responses (such as using home remedies or visiting a pharmacy instead of seeking urgent care) were significantly associated with male sex and lower socioeconomic status (Fig 3). Lower odds of expressing complete agreement with scheduling a medical appointment were observed among men, individuals with lower socioeconomic status, lower incomes, and lower educational attainment. Similarly, lower odds of expressing complete agreement with calling emergency services were found among men, individuals with low educational attainment, and those with lower incomes (Fig 3).

**Fig 3:**
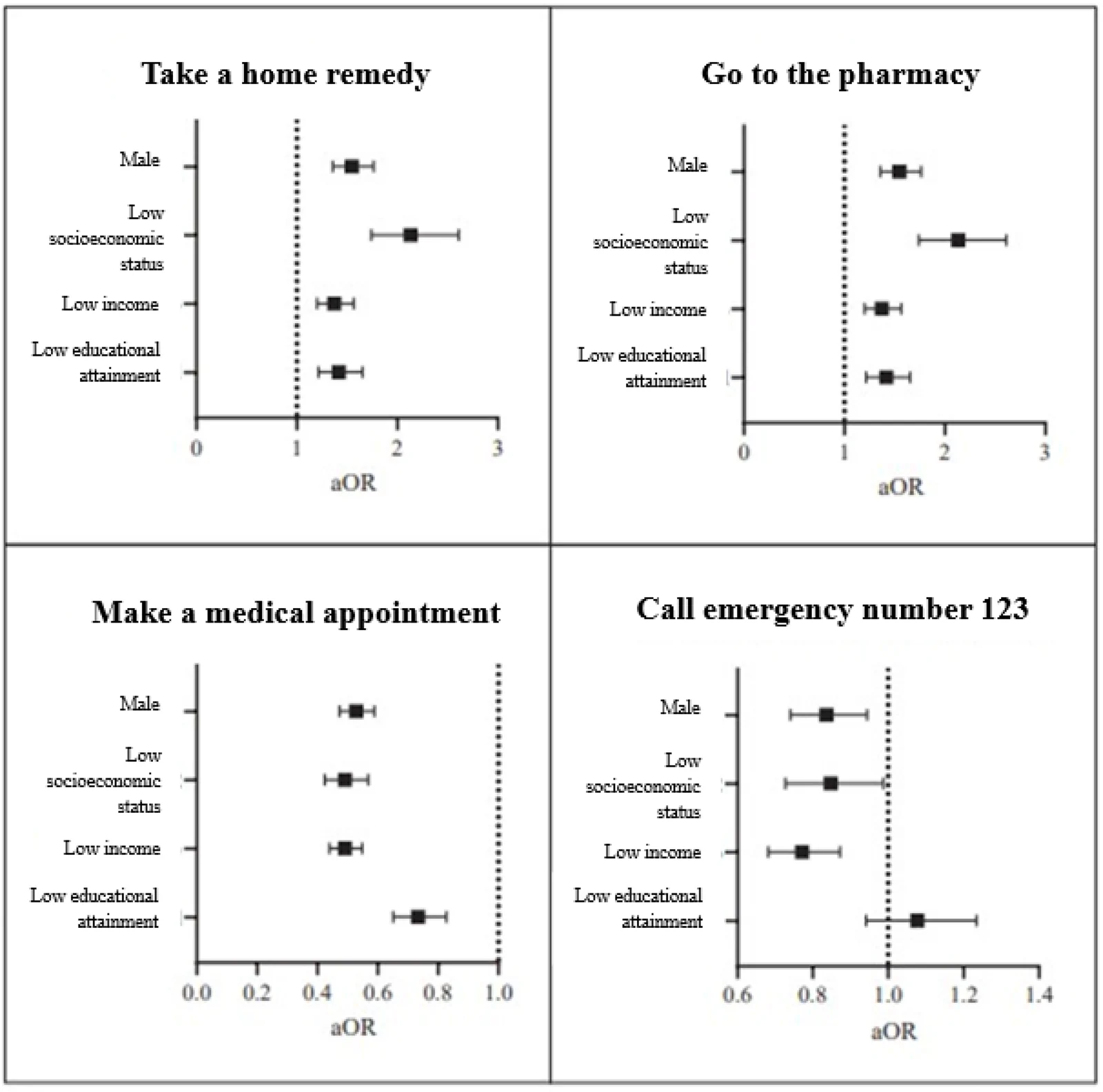
Predictors of Intended Care-seeking Responses to Suspected Stroke. Summary of the estimated association between intended care-seeking stroke practices among participants and sociodemographic variables. The odds ratio (aOR) was adjusted for age and past medical history of systemic arterial hypertension.

## Discussion

In this cross-sectional study of adults with hypertension and type 2 diabetes in Colombia, we identified important gaps in stroke literacy despite high awareness of its severity. While most participants recognized the potential for disability and death, nearly one third lacked knowledge of warning symptoms, and a notable proportion did not completely agree with activating emergency medical services if stroke were suspected. Male sex, lower educational attainment, and lower socioeconomic status were independently associated with limited symptom knowledge and non-emergency intended responses. These patterns suggest that disparities in stroke preparedness persist even among individuals at elevated vascular risk.

Deficits in symptom recognition and intended care-seeking response may translate into delays in hospital presentation, thereby reducing eligibility for time-sensitive reperfusion therapies. Reported thrombolysis rates in Colombia did not exceed 17% in 2019, with potentially lower rates in regions with limited access to reperfusion therapies [18,19]. Although national data in Colombia are limited and largely derived from single-center registries, the available evidence suggests that patient-level factors, such as stroke literacy, may contribute to suboptimal treatment rates. Other health system constraints were not directly assessed in this study and require future measurements.

Evidence from other settings supports the link between stroke literacy and timely care. A systematic review in Africa showed median delays of 31 hours from symptom onset to hospital arrival, largely attributed to poor symptom recognition, failure to seek immediate medical care, and limited transportation access. Similarly, studies in India, Iran, and several African countries have shown that only 8–30% of patients presented within the first 3 hours after symptom onset [20]. Globally, efforts to increase reperfusion treatment rates have focused on physician education, expansion of endovascular therapy access, and strengthening organized stroke systems of care, including prehospital prenotification and coordinated emergency response pathways [21]. However, population-level awareness and stroke literacy remain fundamental components of these strategies. Educational campaigns have been shown to improve stroke knowledge across diverse populations, although evidence on their direct effect on reperfusion therapy uptake is limited [22,23]; nonetheless, enhanced recognition and timely decision-making are associated with shorter onset-to-door times and greater eligibility for acute interventions [24]. Together, these findings underscore that addressing ischemic stroke as a public health priority requires coordinated improvements at both the community and health system levels.

Among the factors influencing stroke awareness in our study, male sex, lower educational attainment, and lower socioeconomic status were independently associated with limited symptom knowledge and non-emergency intended responses, consistent with findings from Lebanon and Indonesia demonstrating the influence of social determinants on stroke literacy [25,26]. Population-based surveys in Uganda and New Zealand have similarly reported significant associations between lower education, lower socioeconomic status, and reduced stroke awareness. Notably, these disparities were observed among individuals already engaged in chronic disease care, suggesting that elevated vascular risk alone does not ensure adequate stroke preparedness. Although treatment delays were not directly measured, these patterns raise concern for persistent inequities in timely access to acute stroke care. In contrast, the role of sex as a predictor of stroke knowledge and intended care-seeking behavior remains inconsistent across settings, with some studies reporting higher awareness among women and others observing gry club beater knowledge among men, indicating that sex-related differences may be context-dependent and shaped by cultural and healthcare factors [27,28].

In Latin America, the EstEPA Project in Argentina, reported that 52.3% of participants would go directly to the emergency department and 39% would activate emergency medical services if stroke were suspected [29]. In contrast, our study revealed a higher proportion (73.4%) reported they would completely agree with calling the emergency hotline. However, more than half also indicated that they would schedule an outpatient appointment, and over one quarter would not activate emergency services. This pattern suggests that although stated willingness to seek urgent care appears comparatively high, uncertainty regarding the appropriate response persists.

Importantly, although most participants in our study recognized that stroke can result in disability (89.6%) and death (91.1%), nearly one third (32.5%) reported no knowledge of warning symptoms. Similar gaps between perceived severity and symptom recognition have been reported in other regions. In southern Saudi Arabia, although 75.6% of respondents demonstrated good general knowledge about stroke, 14% were unable to identify any early warning symptom, while 65.5% identified five or more symptoms [30].

Although health literacy is a critical determinant of timely stroke recognition and care, system-level factors must also be considered within the Colombian healthcare context, particularly the performance and accessibility of emergency medical services (EMS). In our study, nearly 77% of the participants reported that they would call the emergency line if stroke is suspected. This suggests that delays in timely management may not be solely explained by limited awareness, but may also reflect inefficiencies in prehospital triage, transport logistics, or hospital readiness. Understanding these potential system-level barriers through future research could help inform strategies aimed at improving emergency service performance and the quality of stroke care, an area for which local evidence remains scarce. For example, Ro et al. demonstrated in a nationwide Korea cohort that patients transported directly by EMS were significantly more likely to arrive within 2 hours of symptom onset compared with those initially presenting to non-capable hospitals and subsequently transferred [24].

Overall, the present study has several strengths. To our knowledge, based on the extensive literature review conducted, this is the first study in the Colombian population and in Latin America to address the knowledge and perceptions of stroke among individuals at high risk of developing the disease, including those with HTN and DM. This study includes a large sample size collected from various regions across the city, making it the largest known study investigating health literacy about stroke. It employs suitable, robust research methodologies to ensure the reliability and validity of the findings; although results should be interpreted with caution, particularly regarding the internal consistency of the practice domain in the questionnaire and the dichotomization of some variables. Additionally, by focusing on non-health professional individuals, the study provides valuable insights into a segment of the population that is often underrepresented in health literacy research, which will inform and shape public health strategies and educational interventions aimed at improving stroke awareness and prevention. Finally, the study represents a collaborative effort, involving multiple institutions and disciplines, providing a comprehensive representation of Bogotá’s population in Colombia.

This study has some limitations. First, data were self-reported, which may introduce recall bias and social desirability bias, particularly regarding intended emergency behaviors. Second, although the questionnaire was developed through expert review and pilot testing, it had not been previously validated in external populations. While the knowledge domain demonstrated high internal consistency, the practices domain showed low reliability (Cronbach’s α = 0.30), and findings related to intended behaviors should therefore be interpreted with caution. Third, the use of a non-probability sampling strategy limits the representativeness of the sample and may introduce selection bias, particularly given the inclusion of participants recruited from institutional databases and community settings. Consequently, external validity may be limited. Fourth, the cross-sectional design precludes causal inference between sociodemographic factors and stroke knowledge or intended response. Finally, although Bogotá represents a diverse urban population, results may not be generalizable to rural areas or other regions of Colombia with different healthcare infrastructure and socioeconomic distributions.

## Conclusions

This study contributes to a better understanding of stroke literacy among high-risk individuals in Bogotá, Colombia. We identified significant associations between limited recognition of stroke as a medical emergency, socioeconomic disadvantage, and suboptimal intended care-seeking behaviors. This may contribute to inequities in timely access to acute stroke care in our region. These findings highlight the need to strengthen context-specific educational strategies focused on stroke symptom recognition and early decision-making. Addressing both determinants of health and gaps in stroke awareness should be considered a priority component of comprehensive stroke prevention and acute care improvement efforts in Colombia.

## Supporting information

S1 Checklist Inclusivity in global research questionnaire. (DOCX)

S1 File Supplemental materials document. (DOCX)

S1 Data Minimal dataset for stroke health literacy study. (SAV)
