# Supplementary material for: Stroke knowledge and intended care-seeking behavior among adults with hypertension and type 2 diabetes: A cross-sectional study
Source: PLoS One. 2026 Oct 27;21(10):e0335001. doi: 10.1371/journal.pone.0335001 (PMC13712739; doi:10.1371/journal.pone.0335001)
Supplement: Supplementary file 2 [file pone.0335001.s002.docx]

# **Stroke Knowledge and Intended Care-Seeking 2 Behavior Among Adults with Hypertension and Type 2 Diabetes: A Cross-Sectional Study**

## **Supplemental material Table S1. PICO framework**

| **PICO** | **Description** |
| --- | --- |
| Population | Adult patients (aged >18 years) with arterial hypertension and/or diabetes mellitus. |
| Intervention | Implementation of a validated questionnaire that evaluates the knowledge, perceptions, and practices, about ischemic stroke (IS). |
| Comparator | No comparator. |
| Outcome | To determine the knowledge, perceptions, practices, and education regarding ischemic stroke (IS) to inform public health strategies and educational interventions aimed at reducing its burden. |

This PICO framework defines the target population as adult patients with non-communicable chronic conditions, identified as risk factors for stroke. The objective is to implement questionnaires that evaluate their knowledge related to emergency stroke care.

| **Supplemental material Table S2. Sociodemographic characteristics** | | |
| --- | --- | --- |
| **Characteristics** | | **Value** |
| **Age in Years, mean (CI)** | | 66,89 (66,56 - 67,22) |
| **Weight in kilograms, mean (CI)** | | 69,30 (69,00 - 69,59) |
| **Height in meters, mean (CI)** | | 1,60 (1,59 - 1,60) |
| **BMI, mean (CI)** | | 27,15 (27,05 -27,25) |
| **Sex**  **[Frequency (%)]** | **Male** | 2492 (46,30%) |
|  | **Female** | 2887 (53,70%) |
|  | **Other** | 2 (0,01%) |
| **Gender**  **[Frequency (%)]** | **Masculine** | 2483 (46,10%) |
|  | **Feminine** | 2890 (53,70) |
|  | **Non-binary** | 3 (0.10) |
|  | **Prefer not to say** | 3 (0.10) |
|  | **Transgender** | 2 0.05) |
| **Area in Bogotá where participants live**  **[Frequency (%)]** | **Antonio Nariño** | 35 (0.70) |
|  | **Barrios Unidos** | 161 (3,00) |
|  | **Bosa** | 53 (1,00) |
|  | **Chapinero** | 199 (3,70) |
|  | **Ciudad Bolívar** | 954 (17,70) |
|  | **Engativá** | 979 (18,20) |
|  | **Fontibón** | 247 (4,60) |
|  | **Kennedy** | 68 (1,30) |
|  | **La Candelaria** | 27 (0,50) |
|  | **Los Mártires** | 55 (1,00) |
|  | **Puente Aranda** | 16 (0.30) |
|  | **Rafael Uribe Uribe** | 342 (6,40) |
|  | **San Cristóbal** | 359 (6,70) |
|  | **Santa Fe** | 94 (1,70) |
|  | **Suba** | 894 (16,60) |
|  | **Sumapaz** | 13 (0,20) |
|  | **Teusaquillo** | 86 (1,60) |
|  | **Tunjuelito** | 172 (3,20) |
|  | **Usaquén** | 235 (4,40) |
|  | **Usme** | 392 (7,30) |
| **Highest educational level achieved**  **[Frequency (%)]** | **Postgraduate education** | 85 (1,58) |
|  | **Undergraduate education** | 193 (3,59) |
|  | **Primary education** | 2977 (55,32) |
|  | **Secondary education** | 1238 (23,01) |
|  | **No formal education** | 888 (16,5) |
| **Approximate monthly income**  **[Frequency (%)]** | **No income** | 1622 (30,14) |
|  | **Less than $ 500,000 COP** | 1250 (23,23) |
|  | **Between $ 500,000 COP a million COP** | 727 (13,51) |
|  | **Between a million and two million COP** | 1377 (25,59) |
|  | **Between 2 to 5 million COP** | 250 (4,65) |
|  | **Between 5 to 10 million COP** | 86 (1,6) |
|  | **More than 10 million COP** | 68 (1,26) |
| **Socioeconomic stratum**  **[Frequency (%)]** | **0** | 17 (0,32) |
|  | **1** | 1306 (24,28) |
|  | **2** | 3066 (56,99) |
|  | **3** | 770 (14,31) |
|  | **4** | 76 (1,41) |
|  | **5** | 66 (1,23) |
|  | **6** | 79 (1,47) |
| **Marital status [Frequency (%)]** | **Married** | 1528 (28,4) |
|  | **Divorced** | 54 (1) |
|  | **Separated** | 365 (6,78) |
|  | **Single** | 1832 (34,05) |
|  | **Common-law union** | 1124 (20,89) |
|  | **Widowed** | 476 (8,85) |
| **Occupation**  **[Frequency (%)]** | **Study and work** | 16 (0,3) |
|  | **Student** | 8 (0,15) |
|  | **Neither working nor studying** | 3766 (69,99) |
|  | **Working** | 1591 (29,57) |
| **Health insurance regime [Frequency (%)]** | **Contributory** | 363 (6,75) |
|  | **Special** | 37 (0,69) |
|  | **Exception** | 3 (0,06) |
|  | **Does not have a health insurance** | 6 (0,11) |
|  | **Subsidized** | 4972 (92,4) |
| **The confidence interval (CI) was calculated using a 95% confidence level.** | | |

BMI: Body mass index; CI: Confidence interval.
