# Supplementary material for: Stroke knowledge and intended care-seeking behavior among adults with hypertension and type 2 diabetes: A cross-sectional study
Source: PLoS One. 2026 Oct 27;21(10):e0335001. doi: 10.1371/journal.pone.0335001 (PMC13712739; doi:10.1371/journal.pone.0335001)
Supplement: Supplementary file 3 [file pone.0335001.s005.docx]

# **Full title: Stroke Knowledge and Intended Care-Seeking Behavior Among Adults with Hypertension and Type 2 Diabetes: A Cross-Sectional Study**

**Response to Reviewers**

**Edits requested for submission:**

**1. Please note that PLOS ONE is unable to publish previously copyrighted maps or satellite images, or images created using proprietary data. For these reasons, we cannot publish images generated by software which copyrights their output (such as Google Maps, Street View, and Earth). In order to use these images in your submission, we require explicit permission from the copyright owner to publish the figures under the CC BY 4.0 license.**

**At this time, please kindly clarify the following regarding Figure 1:**

**a) Where did the authors obtain the maps, basemaps, shapefiles, map data, etc. in Figure 1?**

**b) Please state whether the maps, basemaps, shapefiles, map data, etc. in Figure 1 have been previously copyrighted to your knowledge.**

**c) If any of the maps, basemaps, shapefiles, map data, etc. in Figure 1 have been previously copyrighted, we require specific consent from the copyright holder to publish these images in PLOS ONE, under the CC BY 4.0 license.**

We appreciate the journal’s guidance regarding the use of copyrighted maps and proprietary data. The figure in question is a modified version of an image originally designed by *Magnific.com*. The figure itself, as we present it here, has not been previously copyrighted. We contacted the company (Magnific) owning the image used as a guide for our Figure 1, and they confirmed that “*The license associated with the resource already grants permission to use the image according to our Terms and Conditions.*” However, they also said that they “[...] *do not provide signed consent forms or formal agreements for publication under the CC BY 4.0 license*.” Proof of this will be uploaded with the attached files as “Other”, showing both the support ticket and the license provided by Magnific.

Accordingly, we are authorized to use the image with proper attribution. The figure has been resubmitted both in the manuscript and in the figures document with the following attribution statement:

*“This figure presents a map of Bogotá illustrating the distribution of the four main health service subnetworks. Among the 5,402 included participants, 27.6% lived in areas covered by the North Subnetwork, 18.7% by the Southwest Subnetwork, 27.8% by the Center-East Subnetwork, and 25.9% by the South Subnetwork. Originally designed by Freepik – Magnific.com (ref X); modified by the authors.”*

We trust this attribution, together with Magnific’s confirmation, satisfies the journal’s requirements for publication under the CC BY 4.0 license.

**2. In the online submission form, you indicated that;**

**"All relevant data are within the manuscript and its Supporting Information files. Data supporting the findings of this study are available from the corresponding author upon reasonable request. Requests will be considered for researchers with a legitimate academic purpose, provided that data use complies with ethical approvals and participant confidentiality. De-identified questionnaire responses can be shared under a data use agreement to ensure privacy."**

**All PLOS journals now require all data underlying the findings described in their manuscript to be freely available to other researchers, either**

**1. In a public repository,**

**2. Within the manuscript itself, or**

**3. Uploaded as supplementary information.**

**This policy applies to all data except where public deposition would breach compliance with the protocol approved by your research ethics board. If your data cannot be made publicly available for ethical or legal reasons (e.g., public availability would compromise patient privacy), please explain your reasons on resubmission and your exemption request will be escalated for approval.**

Thank you for the information. Following the Journal requisites, we have now uploaded the raw data of the study as “Supporting information,” and we indicate that “All relevant data are within the manuscript and its Supporting Information files.”

**Editorial comments:**

When submitting your revision, we need you to address these additional requirements.

1. **Please ensure that your manuscript meets PLOS ONE's style requirements, including those for file naming. The PLOS ONE style templates can be found at** [**https://journals.plos.org/plosone/s/file?id=wjVg/PLOSOne_formatting_sample_main_body.pdf**](https://journals.plos.org/plosone/s/file?id=wjVg/PLOSOne_formatting_sample_main_body.pdf) **and**
   [**https://journals.plos.org/plosone/s/file?id=ba62/PLOSOne_formatting_sample_title_authors_affiliations.pdf**](https://journals.plos.org/plosone/s/file?id=ba62/PLOSOne_formatting_sample_title_authors_affiliations.pdf)
2. **Please include a complete copy of PLOS’ questionnaire on inclusivity in global research in your revised manuscript. Our policy for research in this area aims to improve transparency in the reporting of research performed outside of researchers’ own country or community. The policy applies to researchers who have travelled to a different country to conduct research, research with Indigenous populations or their lands, and research on cultural artefacts. The questionnaire can also be requested at the journal’s discretion for any other submissions, even if these conditions are not met.**

**Please find more information on the policy and a link to download a blank copy of the questionnaire here:** [**https://journals.plos.org/plosone/s/best-practices-in-research-reporting**](https://journals.plos.org/plosone/s/best-practices-in-research-reporting)**.**

**Please upload a completed version of your questionnaire as Supporting Information when you resubmit your manuscript.**

Thank you for the information. We have completed the questionnaire and included it as Supporting Information in our re-submission process.

3. **We note that the grant information you provided in the ‘Funding Information’ and ‘Financial Disclosure’ sections do not match.**

**When you resubmit, please ensure that you provide the correct grant numbers for the awards you received for your study in the ‘Funding Information’ section.**

The award number is **ATENEA-296-2023 (CO1.PCCNTR.5022540)**. This statement has been added in the funding information section accordingly.

4. **Thank you for stating the following financial disclosure: “This study received funding from ATENEA, Agencia Distrital para la Educación Superior, la Ciencia y la Tecnología.“**

**Please state what role the funders took in the study. If the funders had no role, please state: "The funders had no role in study design, data collection and analysis, decision to publish, or preparation of the manuscript.” If this statement is not correct you must amend it as needed.**

**Please include this amended Role of Funder statement in your cover letter; we will change the online submission form on your behalf.**

Thank you for clarifying this. We have added the statement mentioned above regarding the funder’s role and included it in our cover letter.

5. **Please note that funding information should not appear in the Acknowledgments section or other areas of your manuscript. We will only publish funding information present in the Funding Statement section of the online submission form. Please remove any funding-related text from the manuscript.**

Thank you for your comment. We have removed the Funding Statement from the manuscript and included it only in the cover letter and in the appropriate section of the online submission form, as instructed.

6. **Your ethics statement should only appear in the Methods section of your manuscript. If your ethics statement is written in any section besides the Methods, please delete it from any other section.**

Thank you for clarifying this. We have removed the ethics statement from the Acknowledgements section and included it only in the Methods section.

7. **We note that Figure 1 in your submission contain map images which may be copyrighted. All PLOS content is published under the Creative Commons Attribution License (CC BY 4.0), which means that the manuscript, images, and Supporting Information files will be freely available online, and any third party is permitted to access, download, copy, distribute, and use these materials in any way, even commercially, with proper attribution. For these reasons, we cannot publish previously copyrighted maps or satellite images created using proprietary data, such as Google software (Google Maps, Street View, and Earth). For more information, see our copyright guidelines:** [**http://journals.plos.org/plosone/s/licenses-and-copyright**](http://journals.plos.org/plosone/s/licenses-and-copyright)**.

   We require you to either (1) present written permission from the copyright holder to publish these figures specifically under the CC BY 4.0 license, or (2) remove the figures from your submission:**

**You may seek permission from the original copyright holder of Figure 1 to publish the content specifically under the CC BY 4.0 license.

We recommend that you contact the original copyright holder with the Content Permission Form (**[**http://journals.plos.org/plosone/s/file?id=7c09/content-permission-form.pdf**](http://journals.plos.org/plosone/s/file?id=7c09/content-permission-form.pdf)**) and the following text:
“I request permission for the open-access journal PLOS ONE to publish XXX under the Creative Commons Attribution License (CCAL) CC BY 4.0 (**[**http://creativecommons.org/licenses/by/4.0/**](http://creativecommons.org/licenses/by/4.0/)**). Please be aware that this license allows unrestricted use and distribution, even commercially, by third parties. Please reply and provide explicit written permission to publish XXX under a CC BY license and complete the attached form.”

Please upload the completed Content Permission Form or other proof of granted permissions as an ""Other"" file with your submission.

In the figure caption of the copyrighted figure, please include the following text: “Reprinted from [ref] under a CC BY license, with permission from [name of publisher], original copyright [original copyright year].”**

**If you are unable to obtain permission from the original copyright holder to publish these figures under the CC BY 4.0 license or if the copyright holder’s requirements are incompatible with the CC BY 4.0 license, please either i) remove the figure or ii) supply a replacement figure that complies with the CC BY 4.0 license. Please check copyright information on all replacement figures and update the figure caption with source information. If applicable, please specify in the figure caption text when a figure is similar but not identical to the original image and is therefore for illustrative purposes only.
The following resources for replacing copyrighted map figures may be helpful:

USGS National Map Viewer (public domain):** [**http://viewer.nationalmap.gov/viewer/**](http://viewer.nationalmap.gov/viewer/)
**The Gateway to Astronaut Photography of Earth (public domain):** [**http://eol.jsc.nasa.gov/sseop/clickmap/**](http://eol.jsc.nasa.gov/sseop/clickmap/)
**Maps at the CIA (public domain):** [**https://www.cia.gov/library/publications/the-world-factbook/index.html**](https://www.cia.gov/library/publications/the-world-factbook/index.html) **and** [**https://www.cia.gov/library/publications/cia-maps-publications/index.html**](https://www.cia.gov/library/publications/cia-maps-publications/index.html)
**NASA Earth Observatory (public domain):** [**http://earthobservatory.nasa.gov/**](http://earthobservatory.nasa.gov/)
**Landsat:** [**http://landsat.visibleearth.nasa.gov/**](http://landsat.visibleearth.nasa.gov/)
**USGS EROS (Earth Resources Observatory and Science (EROS) Center) (public domain):** [**http://eros.usgs.gov/#**](http://eros.usgs.gov/)
**Natural Earth (public domain):** [**http://www.naturalearthdata.com/**](http://www.naturalearthdata.com/)

Thank you for clarifying this information. After reviewing it, we decided to replace the original image and create our own figure, in which we not only illustrate the distribution of health service subnetworks across the city, but also report the percentages of participants living in the areas covered by each subnetwork. A statement clarifying that the image was created by the authors has been added to the figure legend.

8. **Please include captions for your Supporting Information files at the end of your manuscript, and update any in-text citations to match accordingly. Please see our Supporting Information guidelines for more information:** [**http://journals.plos.org/plosone/s/supporting-information**](http://journals.plos.org/plosone/s/supporting-information)**.**
9. **If the reviewer comments include a recommendation to cite specific previously published works, please review and evaluate these publications to determine whether they are relevant and should be cited. There is no requirement to cite these works unless the editor has indicated otherwise.**

**Reviewer comments:**

**The manuscript addresses a relevant topic; however, substantial revisions are required to improve methodological transparency, conceptual coherence, and accuracy in the presentation of findings.**

**In the abstract, the background information should precede the study objectives to ensure logical flow. Claims regarding questionnaire validation should be tempered, as the practices domain demonstrates very low internal reliability (Cronbach’s α = 0.3). The abstract should explicitly state that a non-probability sampling strategy was used, clearly define “high-risk patients” as individuals with hypertension and/or type 2 diabetes, and specify both the mode of data collection and the analytical/statistical approach employed.**

Thank you for your comment. The abstract has been reorganized so that the background now precedes the objectives. The term “validated” has been revised appropriately throughout the abstract and the main text. Additionally, the requested methodological details have been incorporated into the abstract methods section. All corresponding changes have been made in lines 48–70.

**In the introduction, conceptual coherence should be strengthened by explicitly linking health literacy, prehospital delay, and access to treatment. Terminology regarding the study population must remain consistent throughout, avoiding shifts between the “general population” and patients with hypertension and/or diabetes.**

Thank you for your comments. We have revised the introduction completely to make sure there is better conceptual coherence between presented statistics, concepts, and objectives and relevance of our study. Additionally, the terminologies regarding the study population have been adjusted to keep consistency throughout the entire text.

**In the methods, greater transparency is required regarding the limitations of non-probability sampling and the selection bias introduced by hospital-based recruitment. The methodological justification for restricting the sample to patients with hypertension and/or diabetes should be strengthened. The sample size calculation—based solely on hypertension prevalence—requires clarification of its assumptions and limitations, particularly given the inclusion of patients with diabetes. The reported precision should be framed as an approximate justification rather than a strict inferential guarantee. Given the unacceptable reliability of the practices domain, the instrument should be described as partially validated, and analyses relying on this domain should be interpreted with caution. In addition, the systematic review mentioned in relation to tool development should be included in the reference list, and the personnel responsible for data collection should be clearly identified.**

Thank you for your comments. The limitations of the non-probability sampling approach have now been acknowledged in the sample size determination section (lines 194–197). The rationale for restricting the study population to individuals with diabetes mellitus and hypertension has been thoroughly described in the data collection section (lines 221–226). Additionally, the term “validated” previously used to describe the data collection tool has been reformulated and replaced throughout the abstract and main text. Although a thorough systematic review was conducted, it has not yet been published or submitted for publication. Therefore, we revised the data collection tool section and modified the wording to minimize detailed references to this process, as the review is not publicly available and is not expected to be published in the near future.

**In the results, a table describing the sociodemographic characteristics of participants should be added. Consistency in denominators and clarity regarding numerators and percentages must be ensured. This section should remain strictly descriptive, with interpretative statements moved to the discussion.**

Thank you for your comment. We have now provided a new table containing the sociodemographic characteristics of participants. It is available as supplemental materials table 2. Additionally, numerators and denominators have been reviewed to ensure consistency throughout the article.

**In the discussion, claims of being the “first” study in Latin America should be moderated unless systematically verified. The implications of the low reliability of the practices scale should be more explicitly addressed, and discussion should remain closely aligned with findings directly supported by the data, avoiding unsupported inferences about health system performance.**

Thank you for pointing this out. Prior to conducting this study, we performed an extensive literature review to identify existing evidence that could inform the development of our data collection tool. Although this review has not yet been published, we did not identify studies addressing stroke knowledge and perceptions among populations with vascular risk factors in Colombia or Latin America. Nevertheless, we acknowledge that such statements should be phrased cautiously, and we have revised the wording accordingly to ensure appropriate framing.

The low reliability of the practices scale has now been explicitly acknowledged in the Methodology and Discussion sections. Additionally, the Results and Discussion sections have been thoroughly revised to ensure that observed associations are described in the Results section, while interpretation and analysis of their implications are reserved for the Discussion.

**Finally, the conclusions should be revised to avoid causal language, emphasizing that the findings reflect associations inherent to a cross-sectional design.**

Thank you for your comment. We have revised the conclusion section and improved the presented language accordingly.

**Please use the space provided to explain your answers to the questions above. You may also include additional comments for the author, including concerns about dual publication, research ethics, or publication ethics. (Please upload your review as an attachment if it exceeds 20,000 characters)**

**Reviewer #1**

**Abstract
• In the abstract, the background information should be presented before the study objectives to ensure logical flow and clarity.**

Thank you for pointing this out. The abstract has been reorganized so that the background now precedes the objectives.

**• The abstract states a validated questionnaire, yet the practices domain shows poor internal reliability (Cronbach’s α = 0.3), which contradicts the claim of validation. Temper claims of validation in the abstract.**

Thank you for your comment. The “validated” wording to describe the data collection tool has been reformulated and replaced accordingly throughout the abstract and the text. **Lines 53-61.**

**• The abstract does not clarify that the sample is non-probabilistic, which may mislead readers regarding representativeness. Explicitly state the non-probability sampling approach.**

Thank you for your comment. We have now explicitly clarified that a non-probability sampling strategy was used to recruit the study population. Line 55.

**• The abstract does not specify the mode of data collection (e.g., face-to-face interviews, telephone surveys, or self-administered questionnaires), nor does it describe the analytical approach or statistical tools used.**

Thank you for pointing this out. We have revised the abstract to specify the modes of data collection, including telephone surveys and face-to-face interviews, and to clarify the analytical approach. Lines 58-61.

**• The term “high-risk patients” is used broadly; the abstract should explicitly define this as patients with HTN and/or type 2 DM. Clarify population definition.**

Thank you for your comment. We have now clarified the specific patient population as mentioned above. Lines 50-52.

**Introduction
• Some statistics (e.g., DALYs, preventability) are presented without sufficient contextual linkage to the study population.**

Thank you for pointing this out. We revised the introduction to ensure that the reported statistics are clearly contextualized and directly linked to the study objectives and target population.

**• The introduction occasionally shifts from knowledge gaps to health system capacity (e.g., thrombolysis availability) without a clear conceptual bridge. Improve conceptual coherence by explicitly linking health literacy, prehospital delay, and treatment access.**

Thank you for your comment. We have edited the introduction throughout to ensure better conceptual coherence and overall flow.

**• The final paragraph states “patients diagnosed with DM or HTN,” but earlier sections imply a broader “general population” gap. Maintain consistent terminology regarding the study population.**

Thank you for your comment. The terminology used to describe the study population is now consistent throughout the introduction and text.

**Methods
• The non-probability stratified sampling limits external validity, yet the study frequently implies representativeness. Explicitly acknowledge selection bias in the Methods (not only the Discussion).**

Thank you for your comment. We have now explicitly mentioned the selection bias in the sample size determination section. Lines 172-177.

**• Recruitment via hospital databases excludes high-risk individuals not engaged with healthcare, introducing selection bias. Clarify that findings apply primarily to health-system-engaged, high-risk patients.**

Thank you for pointing this out. To ensure the inclusion of individuals not captured in the institutional registries, complementary recruitment strategies were implemented. These included administering the questionnaire to eligible individuals in community-based settings such as churches, academic events, and public spaces, where participants meeting the inclusion criteria voluntarily agreed to complete the survey. This clarification has now been added to the Data Collection section (lines 212–224).

**• The rationale for including only patients with HTN or DM is not fully justified in methodological terms.**

Thank you for your comment. We have clarified the reasoning for including only patients with the listed risk factors within lines 160-163.

**• Sample size calculation is based solely on HTN prevalence, yet the study includes patients with DM as well. Clarify assumptions and limitations of the sample size estimation.**

Thank you for this observation. The sample size calculation was based on hypertension prevalence because there were reliable population-level projections for hypertension published by Zurique-Sanchez and collaborators, 2019, whereas comparable estimates for diabetes were less robust. Given that both conditions are highly prevalent and strongly associated with stroke risk, we considered hypertension prevalence as a conservative proxy for estimating the required sample size. We have clarified this assumption in the Methods section, and the citation number 13 in the manuscript is the same as the bibliography cited here:

Zurique-Sánchez MS, Zurique-Sánchez CP, López PAC, Sanabria MS, Hernández SCH. Prevalencia de hipertensión arterial en Colombia: Acta Médica Colomb [Internet]. 2019 Sept 30;44(4).

**• Precision (1.18%) is stated without justification for its selection. Reframe the calculation as an approximate justification, not a strict inferential guarantee.**

Thank you for pointing this out. The precision value of 1.18% was not intended as a strict inferential guarantee but rather as an approximate justification of the sample size adequacy (the expected margin of error under the assumptions used). We have revised the text to clarify that this is in lines 177 to 182.

**• The logic of applying population-level prevalence to a non-probabilistic hospital-based sample is weak.**

We acknowledge the reviewer’s concern. Because the study employed a non-probability hospital-based sampling strategy, the sample size calculation based on population-level prevalence should be interpreted as an approximate guide rather than a direct inferential basis. We have revised the Methods to explicitly state this limitation and to emphasize that the findings may not be fully representative of the broader population. This clarification can be found in lines 183 to 185.

**• Cronbach’s alpha of 0.3 for practices is unacceptable, indicating poor internal consistency. Reframe the tool as partially validated.**

Thank you for your comment. The term “validated” has been reframed throughout the abstract and main text, and the questionnaire is now properly described based on the above suggestions.

**• Despite this, practices are used extensively in regression analyses. Acknowledge that findings related to practices should be interpreted with caution.**

Thank you for your comment. We have now acknowledged that the observed poor internal consistency of the practices component of the tool implies that the corresponding results should be interpreted with caution. This clarification has been added to lines 203–206 in the data collection tool section.

**• The questionnaire is described as “validated,” which is misleading. Consider removing or downgrading claims based on the practices scale.**

Thank you for clarifying this. We have removed the term “validated” throughout the abstract and main text and described the questionnaire accordingly based on the above suggestions.

**• The data collection tool mentioned that a systematic review was conducted. Please provide that systematic review in the reference section.**

Although a thorough systematic review was conducted, it has not yet been published or submitted for publication. Therefore, we have revised the data collection tool section and reformulated the wording to limit detailed descriptions of this process, given that the review is not currently publicly available. Lines 187-193.

**• Please clarify who was responsible for data collection, whether trained research assistants, volunteers, or other personnel.**

Thank you for pointing this out. Data collection was conducted by trained nurses, with multiple follow‑ups and audits throughout the process to ensure the reliability of the information obtained. This was now clarified in lines 209-210 in the main manuscript.

**Results**

**• Please provide a table of sociodemographic characteristics of the respondents.**

Thank you for your interest in this data. We have now provided a table with sociodemographic characteristics in the supplemental material (table 2), since we have a limited number of tables allowed to share in the main manuscript. This is specified in lines 285-286.

**• Minor inconsistencies in denominators across tables. Ensure consistency in denominators.**

We thank the reviewer for this observation. We have carefully re-verified all denominators across the tables. We can confirm that they are now correct, specifying the values for every single variable.

**• Some percentages are reported without clear numerators.**

Thank you for this comment. The total values of responders for each variable were reviewed and specified. The total number of responders was specified as the n value at the end of the statements.

**• The Results occasionally interpret findings rather than strictly reporting them. Move interpretative statements to the Discussion.**

Thank you for your comment. We carefully revised the Results and Discussion sections to ensure a clear distinction between reporting and interpretation. While our intention in the Results was to describe the observed associations rather than interpret or analyze their implications, we have adjusted the wording throughout these sections to further improve clarity.

**Discussion**

**• Claims of being the “first” in Latin America may be overstated without systematic verification. Moderate novelty claims.**

Thank you for this comment. Prior to conducting this study, we performed an extensive literature review and did not identify any published studies addressing stroke knowledge and perceptions among populations with vascular risk factors in Colombia or Latin America. To reflect this more cautiously, we have revised the statement in the manuscript in lines 396 to 399.

**• The discussion downplays the serious limitation posed by the low reliability of the practices scale. More explicitly discuss how measurement limitations affect conclusions.**

Thank you for pointing this out. Throughout the discussion, modifications were made to better expose the importance of this study without downplaying the limitations linked to the methodology of the type of study and analysis.

**• Some arguments drift into health system performance without direct data support. Focus discussion tightly on findings directly supported by data.**

Thank you for this valuable comment. Paragraphs between 384 and 395 lines were modified to explain how the results of the study brought these public health matters to the discussion, having in mind the limitations of the results and the main conclusions that could be derived from this.

**Conclusions**

**• Conclusions occasionally imply causality, which is not supported by a cross-sectional design. Rephrase conclusions to emphasize associations rather than causal effects.**

Thank you for addressing this point. Conclusions were rephrased to show the importance of the present investigation without compromising causality in it.

**Reviewer #2**

**- The manuscript describes a technically sound observational study, and the data largely support the authors’ conclusions. The study uses an appropriate cross-sectional design to assess knowledge, perceptions, and practices related to stroke among high-risk patients. The large sample size (n=5,402), inclusion of multiple healthcare subnetworks, and use of multivariable logistic regression support the internal validity of the findings. The statistical analyses are appropriate, and the results are clearly presented with consistent reporting of effect estimates and confidence intervals.**

**The conclusions regarding associations between lower stroke knowledge and male sex, lower education, and lower socioeconomic status are supported by the data. The associations observed between sociodemographic factors and self-reported emergency care practices are also consistent and in line with previous studies. However, some conclusions would benefit from more cautious wording. The cross-sectional design does not allow causal conclusions, and real-world delays in care or clinical outcomes were not directly measured. In addition, the low internal consistency of the practices domain (Cronbach’s alpha = 0.3) limits the strength of conclusions related to behavioral responses. These limitations should be more clearly reflected in the conclusions.**

**Overall, the study meets the technical standards for publication, provided that the interpretation of findings, especially those related to practices, is appropriately cautious.**

**- The statistical analysis is generally appropriate, but only partially rigorous. While suitable methods were used, several key variables, such as socioeconomic status, education, and income, were simplified into binary categories for analytical convenience, which may have reduced analytical precision and obscured potential gradients. The authors should explicitly acknowledge that socioeconomic status, education, and income were dichotomized for analytical simplicity, and note that this approach may have reduced analytical precision and obscured potential gradients in stroke knowledge and practices. No additional analyses are required, but interpretations of these findings should be correspondingly cautious.**

Thank you for this observation. In response, we have updated the Data Analysis section of the methods, explicitly acknowledging that socio -economic status, income, and education were dichotomized for analytical simplicity, and that this approach may have reduced analytical precision and obscured potential gradients in stroke knowledge and practices. This is found in lines 248 to 256.

Moreover, we rephrased some paragraphs in the discussion and the conclusions to better fit the scope and limitations of the study. This can be seen mainly in 411-424.

**- The authors state that all relevant data are included within the manuscript and its Supporting Information. Summary statistics and regression results are clearly reported and sufficient to support the main findings. However, the underlying individual-level data (e.g. anonymized questionnaire responses or datasets used to generate the reported estimates) are not directly provided as supplementary files nor deposited in a public repository. While this may be acceptable given privacy considerations, the data availability could be improved by clarifying whether de-identified raw data can be shared and, if so, under what conditions.**

Thank you for pointing this out. In response to this comment, we have clarified out the data availability (lines 440 to 445). We specify that de-identified raw data will be available for qualified researchers under reasonable request, which includes a legitimate academic purpose and agreement to ensure privacy of the data.

**- The manuscript is generally well organized, intelligible, and written in clear, standard English. The structure follows a logical flow, and the aims, methods, results, and conclusions are easy to follow. The language is appropriate for an international scientific audience, and the main messages are communicated clearly.**

**However, minor grammatical issues, occasional awkward phrasing, and some repetitive or overly long sentences are present, particularly in the Discussion section. These issues do not impede understanding but should be addressed during revision.**

Thank you for your comment. We have thoroughly revised the discussion section to improve grammar and

structure of the sentences, allowing a clearer understanding of the presented ideas.

**Reviewer #3:**

**It is an interesting study conducted for the first time in Columbia. The study captures the larger number of population showing the representativeness. I learn that this study generates evidence for further programs in this area in Columbia and elsewhere.**

Thank you so much for taking the time to review our manuscript. We hope this advances science in stroke and health literacy.
