# Supplementary material for: Stroke knowledge and intended care-seeking behavior among adults with hypertension and type 2 diabetes: A cross-sectional study
Source: PLoS One. 2026 Oct 27;21(10):e0335001. doi: 10.1371/journal.pone.0335001 (PMC13712739; doi:10.1371/journal.pone.0335001)
Supplement: Supplementary file 4 [file pone.0335001.s006.docx]

# **Full title: Stroke Knowledge and Intended Care-Seeking Behavior Among Adults with Hypertension and Type 2 Diabetes: A Cross-Sectional Study**

**Response to Reviewers**

**Edits requested for submission:**

1. **If the reviewer comments include a recommendation to cite specific previously published works, please review and evaluate these publications to determine whether they are relevant and should be cited. There is no requirement to cite these works unless the editor has indicated otherwise.**

Thank you; there were no recommendations to cite published works.

2. **Please review your reference list to ensure that it is complete and correct. If you have cited papers that have been retracted, please include the rationale for doing so in the manuscript text, or remove these references and replace them with relevant current references. Any changes to the reference list should be mentioned in the rebuttal letter that accompanies your revised manuscript. If you need to cite a retracted article, indicate the article’s retracted status in the References list and also include a citation and full reference for the retraction notice.**

Thank you; the reference list was reviewed.

**Reviewer #1**

**Dear Author,

Thank you for carefully revising your manuscript in accordance with the comments and instructions provided. We appreciate the effort you have invested in addressing the revisions.

I am pleased to inform you that your manuscript is now suitable for acceptance. Congratulations on this achievement, and we wish you every success with the publication of your work.**

Thank you so much for taking the time to review our manuscript. We hope this advances science in stroke and health literacy. We will continue working to improve the manuscript and ensure visibility of our findings.

**Reviewer #2**

**Thank you for the revised manuscript. Several previous comments have been addressed. However, the following issues still require correction before the manuscript can be considered for publication.**

**1. Numerical inconsistencies and discrepancies between tables and text

Some numerators, denominators, percentages, and totals reported in the tables do not appear to reconcile. In addition, the direction of some sex-specific findings described in the Results does not correspond to the values shown in the tables.

Please recheck all principal results directly against the original dataset and revise the Abstract, Results, tables, figures, and Discussion so that all values and interpretations are fully consistent.**

We express our gratitude to the reviewer for bringing these critical details to our attention. We have undertaken a complete, systematic audit of our original dataset and carefully rechecked every numerator, denominator, percentage, and summary statistic across the manuscript, including the Abstract, Results, Discussion, tables, and figures, to ensure full reconciliation and accuracy.

Specifically, we have implemented the following revisions:

- **Hypertension Percentages:** We corrected the reported hypertension percentages in lines 292 and 293 of the revised manuscript to align precisely with the raw dataset.
- **Dyslipidemia Terminology:** We updated the label from "Dyslipidemia" to **"Any Dyslipidemia"** throughout the text (line 295) and tables (Table 1) to clearly communicate that this category encompasses both cholesterol and triglyceride abnormalities.
- **Missing Data and Totals:** We explicitly accounted for non-responses in our item-level breakdowns. Specifically, two participants (one male, one female) declined to answer the items regarding calling the emergency hotline, making a medical appointment, and taking a home remedy. Additionally, three participants (two females, one male) declined to answer the item regarding visiting a pharmacy. The overall totals and denominators across our tables now accurately reflect the inclusion of intersex participants as well as those who selected "do not wish to answer." This information was added in the Table 2 footnote.
- **Correction of Sex-Specific Findings:** We are immensely grateful to the reviewer for pointing out the error in lines 315-316 regarding the direction of the sex-specific association. The text previously stated that women were more likely to activate emergency services; this was an error in drafting. The data demonstrate that **men** were more likely than women to intend to call emergency services. We have corrected the text accordingly, and we have reviewed our Discussion section (lines 366–380) to accurately reflect and contextualize this corrected direction.

**2. Incorrect interpretation of the dichotomized Likert responses

The Methods state that “totally agree” was coded as 1 and all other responses were coded as 0. This means that participants who selected “agree” were grouped together with those selecting neutral or disagreement responses.

However, this combined group is described as participants who “would not call emergency services” or who “disagreed” with calling.

Please either present the original Likert response distribution or describe the comparison accurately as “totally agree” versus “all other responses.” Do not interpret the combined group as refusing or disagreeing to call emergency services.**

Thank you for bringing this to our attention. In accordance with the recommendation, we have thoroughly revised the text across the manuscript to describe our comparisons accurately through the lens of complete agreement ("totally agree") versus all other response options combined ("agree," "neutral," "disagree," and "totally disagree"). Specifically, we have implemented these adjustments in:

- **Results Section (Lines 300–332):** Re-framed all descriptions of intended care-seeking behaviors to explicitly present comparisons as "totally agree" versus "all other responses," removing any language asserting that the comparison group disagreed with or refused emergency care.
- **Discussion Section (Lines 337–338, 384):** Updated our interpretation of participant responses to accurately reflect non-complete agreement (highlighting potential hesitation in care-seeking intent) rather than outright refusal or disagreement.

**3. Intended responses should not be described as actual practices

The questionnaire assessed what participants said they would do in a hypothetical stroke situation. It did not measure their actual behavior during a real stroke event.

For example, Table 2 is titled as referring to “practices,” although the study measured intended responses.

Please replace terms such as “practices” and “behavioral responses” with “intended care-seeking responses” or “self-reported intended responses” throughout the manuscript, including table and figure titles.**

Thank you for this comment. In accordance with your recommendation, we have systematically replaced terms such as "practices" and "behavioral responses" with **"intended care-seeking responses"** throughout the manuscript. Specifically, we have updated:

- **Table and Figure Titles:** Revised all titles to accurately specify intended care-seeking responses (including Table 2, Figure 3, and the Figure 3 description). An updated figures document will be uploaded as well.
- **Results Section (Lines 312–329):** Adjusted all narrative descriptions of participant choices to reflect self-reported care-seeking intentions.
- **Discussion Section (Lines 342, 376, and 384):** Refined our interpretations to consistently emphasize that the findings reflect intended, rather than actual, stroke care-seeking behavior.

**4. Analytical sample and denominators

Different denominators are used across the manuscript, but the reason is not clearly explained.

Please define the final analytical sample and state whether each analysis used complete-case or available-case data. The number of participants included in each table and regression model should be clearly reported.**

We appreciate you requesting this important clarification. We have updated the Methods and table footnotes to state that we used an available-case analysis approach and to clarify all denominator variations:

- **Analytical Approach:** Participants were included in each analysis where complete data for the variables of interest were available. Exact values are explicitly reported for every table, figure, and regression model, and the explanation for the missing values was stated in the table’s footnote.
- **Dyslipidemia Sample (N = 5,398):** Differs from the full sample (N = 5,400) because one male participant reported no knowledge of dyslipidemia (n = 2,501) and one female participant declined to answer (n = 2,897).
- **Table 2 Sex Breakdown (N = 5,398):** Sums to 2,502 men and 2,896 women. Two participants reporting other sex identities were retained in the overall total (N = 5,400) but excluded from sex-stratified comparisons due to low frequency (lines 283–284).

We appreciate this feedback, which has enhanced the methodological transparency of our paper.

**5. Conclusions extend beyond the measured outcomes

The manuscript discusses possible problems in emergency medical services, transport, hospital readiness, timely access to care, and treatment delays. These outcomes were not measured.

Please present these issues only as possible topics for future research. The Conclusions should be limited to associations between sociodemographic characteristics, stroke knowledge, and intended care-seeking responses.**

Thank you for this important observation. We agree that our conclusions and discussion must strictly reflect the variables measured in our study.

In accordance with your feedback, we have updated lines 348–349 and line 402 to explicitly state that systemic factors (such as EMS transport dynamics, hospital readiness, and treatment delays) were not directly assessed in this study and represent essential directions for future research. Furthermore, phrasing at the beginning of paragraph 3 (line 350) and line 374 already emphasizes "evidence from other settings," ensuring these contextual links are presented strictly as background context rather than direct findings. Moreover, we eliminated the phrase "timely activation of emergency medical services" from the Conclusions section. The conclusions are now strictly limited to our measured outcomes: the associations between sociodemographic characteristics, stroke knowledge, and self-reported intended care-seeking responses. We appreciate this valuable recommendation, which has strengthened the focus and rigor of our manuscript.

**6. Data Availability statement

The manuscript states that data are available upon reasonable request, while the submission information indicates that the data are included in the Supporting Information. Please make these statements consistent.**

We thank the reviewer for identifying this inconsistency. We have updated the Data Availability statement in the manuscript (line 452) to confirm that the dataset is fully available within the Supporting Information files accompanying the submission.

**7. The manuscript states that surveys were conducted either by telephone or in person, while the ethics statement indicates that written informed consent was obtained from all participants. Please clarify how written consent was obtained from participants interviewed by telephone and confirm that this consent procedure was approved by the relevant ethics committee.**

Thank you for highlighting this discrepancy. The manuscript has been updated to distinguish the consent methods employed. Written informed consent was secured for all face-to-face interviews. For participants surveyed via telephone, verbal informed consent was obtained through a protocol specifically approved by the institutional review boards of UHFSFB and the participating hospitals within the governmental health subnetworks of the North, South, and Center-East (Serial number: SNACEI-132. File 73). These clarifications have been integrated into the Ethical Considerations section of the Methods, lines 272 to 274, to ensure accuracy regarding the approved procedures.
