# Supplementary material for: Stroke knowledge and intended care-seeking behavior among adults with hypertension and type 2 diabetes: A cross-sectional study
Source: PLoS One. 2026 Oct 27;21(10):e0335001. doi: 10.1371/journal.pone.0335001 (PMC13712739; doi:10.1371/journal.pone.0335001)
Supplement: Peer review history

# Peer review: Decision Letter 0

**DOI:** 10.1371/journal.pone.0335001.r001

**Authors:** Oriana Rivera-Lozada de Bonilla

12 Jan 2026

Dear Dr. Alviz,

Thank you for submitting your manuscript to PLOS ONE. After careful consideration, we feel that it has merit but does not fully meet PLOS ONE’s publication criteria as it currently stands. Therefore, we invite you to submit a revised version of the manuscript that addresses the points raised during the review process.

Please submit your revised manuscript by Feb 26 2026 11:59PM. If you will need more time than this to complete your revisions, please reply to this message or contact the journal office at plosone@plos.org. When you're ready to submit your revision, log on to https://www.editorialmanager.com/pone/ and select the 'Submissions Needing Revision' folder to locate your manuscript file.

- A letter that responds to each point raised by the academic editor and reviewer(s). You should upload this letter as a separate file labeled 'Response to Reviewers'.
- A marked-up copy of your manuscript that highlights changes made to the original version. You should upload this as a separate file labeled 'Revised Manuscript with Track Changes'.
- An unmarked version of your revised paper without tracked changes. You should upload this as a separate file labeled 'Manuscript'.

If you would like to make changes to your financial disclosure, please include your updated statement in your cover letter. Guidelines for resubmitting your figure files are available below the reviewer comments at the end of this letter.

If applicable, we recommend that you deposit your laboratory protocols in protocols.io to enhance the reproducibility of your results. Protocols.io assigns your protocol its own identifier (DOI) so that it can be cited independently in the future. For instructions see: https://journals.plos.org/plosone/s/submission-guidelines#loc-laboratory-protocols. Additionally, PLOS ONE offers an option for publishing peer-reviewed Lab Protocol articles, which describe protocols hosted on protocols.io. Read more information on sharing protocols at https://plos.org/protocols?utm_medium=editorial-email&utm_source=authorletters&utm_campaign=protocols.

We look forward to receiving your revised manuscript.

Kind regards,

Oriana Rivera-Lozada de Bonilla

Academic Editor

PLOS One

Journal Requirements:

When submitting your revision, we need you to address these additional requirements.

1. Please ensure that your manuscript meets PLOS ONE's style requirements, including those for file naming. The PLOS ONE style templates can be found at

https://journals.plos.org/plosone/s/file?id=wjVg/PLOSOne_formatting_sample_main_body.pdf and

https://journals.plos.org/plosone/s/file?id=ba62/PLOSOne_formatting_sample_title_authors_affiliations.pdf

2. Please include a complete copy of PLOS’ questionnaire on inclusivity in global research in your revised manuscript. Our policy for research in this area aims to improve transparency in the reporting of research performed outside of researchers’ own country or community. The policy applies to researchers who have travelled to a different country to conduct research, research with Indigenous populations or their lands, and research on cultural artefacts. The questionnaire can also be requested at the journal’s discretion for any other submissions, even if these conditions are not met.

Please find more information on the policy and a link to download a blank copy of the questionnaire here: https://journals.plos.org/plosone/s/best-practices-in-research-reporting.

Please upload a completed version of your questionnaire as Supporting Information when you resubmit your manuscript.

3. We note that the grant information you provided in the ‘Funding Information’ and ‘Financial Disclosure’ sections do not match.

When you resubmit, please ensure that you provide the correct grant numbers for the awards you received for your study in the ‘Funding Information’ section.

4. Thank you for stating the following financial disclosure:

“This study received funding from ATENEA, Agencia Distrital para la Educación Superior, la Ciencia y la Tecnología.“

Please state what role the funders took in the study.  If the funders had no role, please state: "The funders had no role in study design, data collection and analysis, decision to publish, or preparation of the manuscript.” If this statement is not correct you must amend it as needed.

Please include this amended Role of Funder statement in your cover letter; we will change the online submission form on your behalf.

5. Please note that funding information should not appear in the Acknowledgments section or other areas of your manuscript. We will only publish funding information present in the Funding Statement section of the online submission form. Please remove any funding-related text from the manuscript.

6. Your ethics statement should only appear in the Methods section of your manuscript. If your ethics statement is written in any section besides the Methods, please delete it from any other section.

7. We note that Figure 1 in your submission contain map images which may be copyrighted. All PLOS content is published under the Creative Commons Attribution License (CC BY 4.0), which means that the manuscript, images, and Supporting Information files will be freely available online, and any third party is permitted to access, download, copy, distribute, and use these materials in any way, even commercially, with proper attribution. For these reasons, we cannot publish previously copyrighted maps or satellite images created using proprietary data, such as Google software (Google Maps, Street View, and Earth). For more information, see our copyright guidelines: http://journals.plos.org/plosone/s/licenses-and-copyright.

We require you to either (1) present written permission from the copyright holder to publish these figures specifically under the CC BY 4.0 license, or (2) remove the figures from your submission:

1) You may seek permission from the original copyright holder of Figure 1 to publish the content specifically under the CC BY 4.0 license.

We recommend that you contact the original copyright holder with the Content Permission Form (http://journals.plos.org/plosone/s/file?id=7c09/content-permission-form.pdf) and the following text:

“I request permission for the open-access journal PLOS ONE to publish XXX under the Creative Commons Attribution License (CCAL) CC BY 4.0 (http://creativecommons.org/licenses/by/4.0/). Please be aware that this license allows unrestricted use and distribution, even commercially, by third parties. Please reply and provide explicit written permission to publish XXX under a CC BY license and complete the attached form.”

Please upload the completed Content Permission Form or other proof of granted permissions as an ""Other"" file with your submission.

In the figure caption of the copyrighted figure, please include the following text: “Reprinted from [ref] under a CC BY license, with permission from [name of publisher], original copyright [original copyright year].”

2) If you are unable to obtain permission from the original copyright holder to publish these figures under the CC BY 4.0 license or if the copyright holder’s requirements are incompatible with the CC BY 4.0 license, please either i) remove the figure or ii) supply a replacement figure that complies with the CC BY 4.0 license. Please check copyright information on all replacement figures and update the figure caption with source information. If applicable, please specify in the figure caption text when a figure is similar but not identical to the original image and is therefore for illustrative purposes only.

The following resources for replacing copyrighted map figures may be helpful:

USGS National Map Viewer (public domain): http://viewer.nationalmap.gov/viewer/

The Gateway to Astronaut Photography of Earth (public domain): http://eol.jsc.nasa.gov/sseop/clickmap/

Maps at the CIA (public domain): https://www.cia.gov/library/publications/the-world-factbook/index.html and https://www.cia.gov/library/publications/cia-maps-publications/index.html

NASA Earth Observatory (public domain): http://earthobservatory.nasa.gov/

Landsat: http://landsat.visibleearth.nasa.gov/

USGS EROS (Earth Resources Observatory and Science (EROS) Center) (public domain): http://eros.usgs.gov/#

Natural Earth (public domain): http://www.naturalearthdata.com/

8. Please include captions for your Supporting Information files at the end of your manuscript, and update any in-text citations to match accordingly. Please see our Supporting Information guidelines for more information: http://journals.plos.org/plosone/s/supporting-information.

9. If the reviewer comments include a recommendation to cite specific previously published works, please review and evaluate these publications to determine whether they are relevant and should be cited. There is no requirement to cite these works unless the editor has indicated otherwise.

**Additional Editor Comments:**

The manuscript addresses a relevant topic; however, substantial revisions are required to improve methodological transparency, conceptual coherence, and accuracy in the presentation of findings.

In the **abstract** , the background information should precede the study objectives to ensure logical flow. Claims regarding questionnaire validation should be tempered, as the practices domain demonstrates very low internal reliability (Cronbach’s α = 0.3). The abstract should explicitly state that a non-probability sampling strategy was used, clearly define “high-risk patients” as individuals with hypertension and/or type 2 diabetes, and specify both the mode of data collection and the analytical/statistical approach employed.

In the **introduction** , conceptual coherence should be strengthened by explicitly linking health literacy, prehospital delay, and access to treatment. Terminology regarding the study population must remain consistent throughout, avoiding shifts between the “general population” and patients with hypertension and/or diabetes.

In the **methods** , greater transparency is required regarding the limitations of non-probability sampling and the selection bias introduced by hospital-based recruitment. The methodological justification for restricting the sample to patients with hypertension and/or diabetes should be strengthened. The sample size calculation—based solely on hypertension prevalence—requires clarification of its assumptions and limitations, particularly given the inclusion of patients with diabetes. The reported precision should be framed as an approximate justification rather than a strict inferential guarantee. Given the unacceptable reliability of the practices domain, the instrument should be described as partially validated, and analyses relying on this domain should be interpreted with caution. In addition, the systematic review mentioned in relation to tool development should be included in the reference list, and the personnel responsible for data collection should be clearly identified.

In the **results** , a table describing the sociodemographic characteristics of participants should be added. Consistency in denominators and clarity regarding numerators and percentages must be ensured. This section should remain strictly descriptive, with interpretative statements moved to the discussion.

In the **discussion** , claims of being the “first” study in Latin America should be moderated unless systematically verified. The implications of the low reliability of the practices scale should be more explicitly addressed, and discussion should remain closely aligned with findings directly supported by the data, avoiding unsupported inferences about health system performance.

Finally, the **conclusions**  should be revised to avoid causal language, emphasizing that the findings reflect associations inherent to a cross-sectional design.

Reviewer's Responses to Questions

**Comments to the Author**

1. Is the manuscript technically sound, and do the data support the conclusions?

Reviewer #1: Yes

Reviewer #2: Yes

Reviewer #3: Yes

**********

2. Has the statistical analysis been performed appropriately and rigorously?

Reviewer #1: Yes

Reviewer #2: Yes

Reviewer #3: I Don't Know

**********

3. Have the authors made all data underlying the findings in their manuscript fully available?

The PLOS Data policy

Reviewer #1: Yes

Reviewer #2: Yes

Reviewer #3: Yes

**********

4. Is the manuscript presented in an intelligible fashion and written in standard English?

Reviewer #1: Yes

Reviewer #2: Yes

Reviewer #3: Yes

**********

Reviewer #1: Abstract

• In the abstract, the background information should be presented before the study objectives to ensure logical flow and clarity.

• The abstract states a validated questionnaire, yet the practices domain shows poor internal reliability (Cronbach’s α = 0.3), which contradicts the claim of validation. Temper claims of validation in the abstract.

• The abstract does not clarify that the sample is non-probabilistic, which may mislead readers regarding representativeness. Explicitly state the non-probability sampling approach.

• The abstract does not specify the mode of data collection (e.g., face-to-face interviews, telephone surveys, or self-administered questionnaires), nor does it describe the analytical approach or statistical tools used.

• The term “high-risk patients” is used broadly; the abstract should explicitly define this as patients with HTN and/or type 2 DM. Clarify population definition.

Introduction

• Some statistics (e.g., DALYs, preventability) are presented without sufficient contextual linkage to the study population.

• The introduction occasionally shifts from knowledge gaps to health system capacity (e.g., thrombolysis availability) without a clear conceptual bridge. Improve conceptual coherence by explicitly linking health literacy, prehospital delay, and treatment access.

• The final paragraph states “patients diagnosed with DM or HTN,” but earlier sections imply a broader “general population” gap. Maintain consistent terminology regarding the study population.

Methods

• The non-probability stratified sampling limits external validity, yet the study frequently implies representativeness. Explicitly acknowledge selection bias in the Methods (not only the Discussion).

• Recruitment via hospital databases excludes high-risk individuals not engaged with healthcare, introducing selection bias. Clarify that findings apply primarily to health-system-engaged, high-risk patients.

• The rationale for including only patients with HTN or DM is not fully justified in methodological terms.

• Sample size calculation is based solely on HTN prevalence, yet the study includes patients with DM as well. Clarify assumptions and limitations of the sample size estimation.

• Precision (1.18%) is stated without justification for its selection. Reframe the calculation as an approximate justification, not a strict inferential guarantee.

• The logic of applying population-level prevalence to a non-probabilistic hospital-based sample is weak.

• Cronbach’s alpha of 0.3 for practices is unacceptable, indicating poor internal consistency. Reframe the tool as partially validated.

• Despite this, practices are used extensively in regression analyses. Acknowledge that findings related to practices should be interpreted with caution.

• The questionnaire is described as “validated,” which is misleading. Consider removing or downgrading claims based on the practices scale.

• The data collection tool mentioned that a systematic review was conducted. Please provide that systematic review in the reference section.

• Please clarify who was responsible for data collection, whether trained research assistants, volunteers, or other personnel.

Results

• Please provide a table of sociodemographic characteristics of the respondents.

• Minor inconsistencies in denominators across tables. Ensure consistency in denominators.

• Some percentages are reported without clear numerators.

• The Results occasionally interpret findings rather than strictly reporting them. Move interpretative statements to the Discussion.

Discussion

• Claims of being the “first” in Latin America may be overstated without systematic verification. Moderate novelty claims.

• The discussion downplays the serious limitation posed by the low reliability of the practices scale. More explicitly discuss how measurement limitations affect conclusions.

• Some arguments drift into health system performance without direct data support. Focus discussion tightly on findings directly supported by data.

Conclusions

• Conclusions occasionally imply causality, which is not supported by a cross-sectional design. Rephrase conclusions to emphasize associations rather than causal effects.

Reviewer #2: - The manuscript describes a technically sound observational study, and the data largely support the authors’ conclusions.

The study uses an appropriate cross-sectional design to assess knowledge, perceptions, and practices related to stroke among high-risk patients. The large sample size (n=5,402), inclusion of multiple healthcare subnetworks, and use of multivariable logistic regression support the internal validity of the findings. The statistical analyses are appropriate, and the results are clearly presented with consistent reporting of effect estimates and confidence intervals.

The conclusions regarding associations between lower stroke knowledge and male sex, lower education, and lower socioeconomic status are supported by the data. The associations observed between sociodemographic factors and self-reported emergency care practices are also consistent and in line with previous studies.

However, some conclusions would benefit from more cautious wording. The cross-sectional design does not allow causal conclusions, and real-world delays in care or clinical outcomes were not directly measured. In addition, the low internal consistency of the practices domain (Cronbach’s alpha = 0.3) limits the strength of conclusions related to behavioral responses. These limitations should be more clearly reflected in the conclusions.

Overall, the study meets the technical standards for publication, provided that the interpretation of findings, especially those related to practices, is appropriately cautious.-

- The statistical analysis is generally appropriate, but only partially rigorous. While suitable methods were used, several key variables such as socioeconomic status, education, and income were simplified into binary categories for analytical convenience, which may have reduced analytical precision and obscured potential gradients. The authors should explicitly acknowledge that socioeconomic status, education, and income were dichotomized for analytical simplicity, and note that this approach may have reduced analytical precision and obscured potential gradients in stroke knowledge and practices. No additional analyses are required, but interpretations of these findings should be correspondingly cautious.

- The authors state that all relevant data are included within the manuscript and its Supporting Information. Summary statistics and regression results are clearly reported and sufficient to support the main findings. However, the underlying individual-level data (e.g. anonymized questionnaire responses or datasets used to generate the reported estimates) are not directly provided as supplementary files nor deposited in a public repository. While this may be acceptable given privacy considerations, the data availability could be improved by clarifying whether de-identified raw data can be shared and, if so, under what conditions.

- The manuscript is generally well organized, intelligible, and written in clear, standard English. The structure follows a logical flow, and the aims, methods, results, and conclusions are easy to follow. The language is appropriate for an international scientific audience, and the main messages are communicated clearly.

However, minor grammatical issues, occasional awkward phrasing, and some repetitive or overly long sentences are present, particularly in the Discussion section. These issues do not impede understanding but should be addressed during revision.

Reviewer #3: It is an interesting study conducted for the first time in Columbia. The study captures the larger number of population showing the representativeness. I learn that this study generates evidence for further programs in this area in Columbia and elsewhere.

**********

what does this mean?). If published, this will include your full peer review and any attached files.

If you choose “no”, your identity will remain anonymous but your review may still be made public.

**Do you want your identity to be public for this peer review?**  For information about this choice, including consent withdrawal, please see our Privacy Policy

Reviewer #1: **Yes:**  Faizul Akmal Abdul Rahim

Reviewer #2: No

Reviewer #3: No

**********

[NOTE: If reviewer comments were submitted as an attachment file, they will be attached to this email and accessible via the submission site. Please log into your account, locate the manuscript record, and check for the action link "View Attachments". If this link does not appear, there are no attachment files.]

To ensure your figures meet our technical requirements, please review our figure guidelines: https://journals.plos.org/plosone/s/figures

You may also use PLOS’s free figure tool, NAAS, to help you prepare publication quality figures: https://journals.plos.org/plosone/s/figures#loc-tools-for-figure-preparation.

NAAS will assess whether your figures meet our technical requirements by comparing each figure against our figure specifications.

# Author comment: Author response to Decision Letter 1

**DOI:** 10.1371/journal.pone.0335001.r002

8 Jun 2026

Editorial comments:

When submitting your revision, we need you to address these additional requirements.

1. Please ensure that your manuscript meets PLOS ONE's style requirements, including those for file naming. The PLOS ONE style templates can be found at https://journals.plos.org/plosone/s/file?id=wjVg/PLOSOne_formatting_sample_main_body.pdf and

https://journals.plos.org/plosone/s/file?id=ba62/PLOSOne_formatting_sample_title_authors_affiliations.pdf

2. Please include a complete copy of PLOS’ questionnaire on inclusivity in global research in your revised manuscript. Our policy for research in this area aims to improve transparency in the reporting of research performed outside of researchers’ own country or community. The policy applies to researchers who have travelled to a different country to conduct research, research with Indigenous populations or their lands, and research on cultural artefacts. The questionnaire can also be requested at the journal’s discretion for any other submissions, even if these conditions are not met.

Please find more information on the policy and a link to download a blank copy of the questionnaire here: https://journals.plos.org/plosone/s/best-practices-in-research-reporting.

Please upload a completed version of your questionnaire as Supporting Information when you resubmit your manuscript.

Thank you for the information. We have completed the questionnaire and included it as Supporting Information in our re-submission process.

3. We note that the grant information you provided in the ‘Funding Information’ and ‘Financial Disclosure’ sections do not match.

When you resubmit, please ensure that you provide the correct grant numbers for the awards you received for your study in the ‘Funding Information’ section.

The award number is ATENEA-296-2023 (CO1.PCCNTR.5022540). This statement has been added in the funding information section accordingly.

4. Thank you for stating the following financial disclosure: “This study received funding from ATENEA, Agencia Distrital para la Educación Superior, la Ciencia y la Tecnología.“

Please state what role the funders took in the study. If the funders had no role, please state: "The funders had no role in study design, data collection and analysis, decision to publish, or preparation of the manuscript.” If this statement is not correct you must amend it as needed.

Please include this amended Role of Funder statement in your cover letter; we will change the online submission form on your behalf.

Thank you for clarifying this. We have added the statement mentioned above regarding the funder’s role and included it in our cover letter.

5. Please note that funding information should not appear in the Acknowledgments section or other areas of your manuscript. We will only publish funding information present in the Funding Statement section of the online submission form. Please remove any funding-related text from the manuscript.

Thank you for your comment. We have removed the Funding Statement from the manuscript and included it only in the cover letter and in the appropriate section of the online submission form, as instructed.

6. Your ethics statement should only appear in the Methods section of your manuscript. If your ethics statement is written in any section besides the Methods, please delete it from any other section.

Thank you for clarifying this. We have removed the ethics statement from the Acknowledgements section and included it only in the Methods section.

7. We note that Figure 1 in your submission contain map images which may be copyrighted. All PLOS content is published under the Creative Commons Attribution License (CC BY 4.0), which means that the manuscript, images, and Supporting Information files will be freely available online, and any third party is permitted to access, download, copy, distribute, and use these materials in any way, even commercially, with proper attribution. For these reasons, we cannot publish previously copyrighted maps or satellite images created using proprietary data, such as Google software (Google Maps, Street View, and Earth). For more information, see our copyright guidelines: http://journals.plos.org/plosone/s/licenses-and-copyright.

We require you to either (1) present written permission from the copyright holder to publish these figures specifically under the CC BY 4.0 license, or (2) remove the figures from your submission:

You may seek permission from the original copyright holder of Figure 1 to publish the content specifically under the CC BY 4.0 license.

We recommend that you contact the original copyright holder with the Content Permission Form (http://journals.plos.org/plosone/s/file?id=7c09/content-permission-form.pdf) and the following text:

“I request permission for the open-access journal PLOS ONE to publish XXX under the Creative Commons Attribution License (CCAL) CC BY 4.0 (http://creativecommons.org/licenses/by/4.0/). Please be aware that this license allows unrestricted use and distribution, even commercially, by third parties. Please reply and provide explicit written permission to publish XXX under a CC BY license and complete the attached form.”

Please upload the completed Content Permission Form or other proof of granted permissions as an ""Other"" file with your submission.

In the figure caption of the copyrighted figure, please include the following text: “Reprinted from [ref] under a CC BY license, with permission from [name of publisher], original copyright [original copyright year].”

If you are unable to obtain permission from the original copyright holder to publish these figures under the CC BY 4.0 license or if the copyright holder’s requirements are incompatible with the CC BY 4.0 license, please either i) remove the figure or ii) supply a replacement figure that complies with the CC BY 4.0 license. Please check copyright information on all replacement figures and update the figure caption with source information. If applicable, please specify in the figure caption text when a figure is similar but not identical to the original image and is therefore for illustrative purposes only.

The following resources for replacing copyrighted map figures may be helpful:

USGS National Map Viewer (public domain): http://viewer.nationalmap.gov/viewer/

The Gateway to Astronaut Photography of Earth (public domain): http://eol.jsc.nasa.gov/sseop/clickmap/

Maps at the CIA (public domain): https://www.cia.gov/library/publications/the-world-factbook/index.html and https://www.cia.gov/library/publications/cia-maps-publications/index.html

NASA Earth Observatory (public domain): http://earthobservatory.nasa.gov/

Landsat: http://landsat.visibleearth.nasa.gov/

USGS EROS (Earth Resources Observatory and Science (EROS) Center) (public domain): http://eros.usgs.gov/#

Natural Earth (public domain): http://www.naturalearthdata.com/

Thank you for clarifying this information. After reviewing it, we decided to replace the original image and create our own figure, in which we not only illustrate the distribution of health service subnetworks across the city, but also report the percentages of participants living in the areas covered by each subnetwork. A statement clarifying that the image was created by the authors has been added to the figure legend.

8. Please include captions for your Supporting Information files at the end of your manuscript, and update any in-text citations to match accordingly. Please see our Supporting Information guidelines for more information: http://journals.plos.org/plosone/s/supporting-information.

9. If the reviewer comments include a recommendation to cite specific previously published works, please review and evaluate these publications to determine whether they are relevant and should be cited. There is no requirement to cite these works unless the editor has indicated otherwise.

Reviewer comments:

The manuscript addresses a relevant topic; however, substantial revisions are required to improve methodological transparency, conceptual coherence, and accuracy in the presentation of findings.

In the abstract, the background information should precede the study objectives to ensure logical flow. Claims regarding questionnaire validation should be tempered, as the practices domain demonstrates very low internal reliability (Cronbach’s α = 0.3). The abstract should explicitly state that a non-probability sampling strategy was used, clearly define “high-risk patients” as individuals with hypertension and/or type 2 diabetes, and specify both the mode of data collection and the analytical/statistical approach employed.

Thank you for your comment. The abstract has been reorganized so that the background now precedes the objectives. The term “validated” has been revised appropriately throughout the abstract and the main text. Additionally, the requested methodological details have been incorporated into the abstract methods section. All corresponding changes have been made in lines 48–70.

In the introduction, conceptual coherence should be strengthened by explicitly linking health literacy, prehospital delay, and access to treatment. Terminology regarding the study population must remain consistent throughout, avoiding shifts between the “general population” and patients with hypertension and/or diabetes.

Thank you for your comments. We have revised the introduction completely to make sure there is better conceptual coherence between presented statistics, concepts, and objectives and relevance of our study. Additionally, the terminologies regarding the study population have been adjusted to keep consistency throughout the entire text.

In the methods, greater transparency is required regarding the limitations of non-probability sampling and the selection bias introduced by hospital-based recruitment. The methodological justification for restricting the sample to patients with hypertension and/or diabetes should be strengthened. The sample size calculation—based solely on hypertension prevalence—requires clarification of its assumptions and limitations, particularly given the inclusion of patients with diabetes. The reported precision should be framed as an approximate justification rather than a strict inferential guarantee. Given the unacceptable reliability of the practices domain, the instrument should be described as partially validated, and analyses relying on this domain should be interpreted with caution. In addition, the systematic review mentioned in relation to tool development should be included in the reference list, and the personnel responsible for data collection should be clearly identified.

Thank you for your comments. The limitations of the non-probability sampling approach have now been acknowledged in the sample size determination section (lines 194–197). The rationale for restricting the study population to individuals with diabetes mellitus and hypertension has been thoroughly described in the data collection section (lines 221–226). Additionally, the term “validated” previously used to describe the data collection tool has been reformulated and replaced throughout the abstract and main text. Although a thorough systematic review was conducted, it has not yet been published or submitted for publication. Therefore, we revised the data collection tool section and modified the wording to minimize detailed references to this process, as the review is not publicly available and is not expected to be published in the near future.

In the results, a table describing the sociodemographic characteristics of participants should be added. Consistency in denominators and clarity regarding numerators and percentages must be ensured. This section should remain strictly descriptive, with interpretative statements moved to the discussion.

Thank you for your comment. We have now provided a new table containing the sociodemographic characteristics of participants. It is available as supplemental materials table 2. Additionally, numerators and denominators have been reviewed to ensure consistency throughout the article.

In the discussion, claims of being the “first” study in Latin America should be moderated unless systematically verified. The implications of the low reliability of the practices scale should be more explicitly addressed, and discussion should remain closely aligned with findings directly supported by the data, avoiding unsupported inferences about health system performance.

Thank you for pointing this out. Prior to conducting this study, we performed an extensive literature review to identify existing evidence that could inform the development of our data collection tool. Although this review has not yet been published, we did not identify studies addressing stroke knowledge and perceptions among populations with vascular risk factors in Colombia or Latin America. Nevertheless, we acknowledge that such statements should be phrased cautiously, and we have revised the wording accordingly to ensure appropriate framing.

The low reliability of the practices scale has now been explicitly acknowledged in the Methodology and Discussion sections. Additionally, the Results and Discussion sections have been thoroughly revised to ensure that observed associations are described in the Results section, while interpretation and analysis of their implications are reserved for the Discussion.

Finally, the conclusions should be revised to avoid causal language, emphasizing that the findings reflect associations inherent to a cross-sectional design.

Thank you for your comment. We have revised the conclusion section and improved the presented language accordingly.

Please use the space provided to explain your answers to the questions above. You may also include additional comments for the author, including concerns about dual publication, research ethics, or publication ethics. (Please upload your review as an attachment if it exceeds 20,000 characters)

Reviewer #1

Abstract

• In the abstract, the background information should be presented before the study objectives to ensure logical flow and clarity.

Thank you for pointing this out. The abstract has been reorganized so that the background now precedes the objectives.

• The abstract states a validated questionnaire, yet the practices domain shows poor internal reliability (Cronbach’s α = 0.3), which contradicts the claim of validation. Temper claims of validation in the abstract.

Thank you for your comment. The “validated” wording to describe the data collection tool has been reformulated and replaced accordingly throughout the abstract and the text. Lines 53-61.

• The abstract does not clarify that the sample is non-probabilistic, which may mislead readers regarding representativeness. Explicitly state the non-probability sampling approach.

Thank you for your comment. We have now explicitly clarified that a non-probability sampling strategy was used to recruit the study population. Line 55.

• The abstract does not specify the mode of data collection (e.g., face-to-face interviews, telephone surveys, or self-administered questionnaires), nor does it describe the analytical approach or statistical tools used.

Thank you for pointing this out. We have revised the abstract to specify the modes of data collection, including telephone surveys and face-to-face interviews, and to clarify the analytical approach. Lines 58-61.

• The term “high-risk patients” is used broadly; the abstract should explicitly define this as patients with HTN and/or type 2 DM. Clarify population definition.

Thank you for your comment. We have now clarified the specific patient population as mentioned above. Lines 50-52.

Introduction

• Some statistics (e.g., DALYs, preventability) are presented without sufficient contextual linkage to the study population.

Thank you for pointing this out. We revised the introduction to ensure that the reported statistics are clearly contextualized and directly linked to the study objectives and target population.

• The introduction occasionally shifts from knowledge gaps to health system capacity (e.g., thrombolysis availability) without a clear conceptual bridge. Improve conceptual coherence by explicitly linking health literacy, prehospital delay, and treatment access.

Thank you for your comment. We have edited the introduction throughout to ensure better conceptual coherence and overall flow.

• The final paragraph states “patients diagnosed with DM or HTN,” but earlier sections imply a broader “general population” gap. Maintain consistent terminology regarding the study population.

Thank you for your comment. The terminology used to describe the study population is now consistent throughout the introduction and text.

Methods

• The non-probability stratified sampling limits external validity, yet the study frequently implies representativeness. Explicitly acknowledge selection bias in the Methods (not only the Discussion).

Thank you for your comment. We have now explicitly mentioned the selection bias in the sample size determination section. Lines 172-177.

• Recruitment via hospital databases excludes high-risk individuals not engaged with healthcare, introducing selection bias. Clarify that findings apply primarily to health-system-engaged, high-risk patients.

Thank you for pointing this out. To ensure the inclusion of individuals not captured in the institutional registries, complementary recruitment strategies were implemented. These included administering the questionnaire to eligible individuals in community-based settings such as churches, academic events, and public spaces, where participants meeting the inclusion criteria voluntarily agreed to complete the survey. This clarification has now been added to the Data Collection section (lines 212–224).

• The rationale for including only patients with HTN or DM is not fully justified in methodological terms.

Thank you for your comment. We have clarified the reasoning for including only patients with the listed risk factors within lines 160-163.

• Sample size calculation is based solely on HTN prevalence, yet the study includes patients with DM as well. Clarify assumptions and limitations of the sample size estimation.

Thank you for this observation. The sample size calculation was based on hypertension prevalence because there were reliable population-level projections for hypertension published by Zurique-Sanchez and collaborators, 2019, whereas comparable estimates for diabetes were less robust. Given that both conditions are highly prevalent and strongly associated with stroke risk, we considered hypertension prevalence as a conservative proxy for estimating the required sample size. We have clarified this assumption in the Methods section, and the citation number 13 in the manuscript is the same as the bibliography cited here:

Zurique-Sánchez MS, Zurique-Sánchez CP, López PAC, Sanabria MS, Hernández SCH. Prevalencia de hipertensión arterial en Colombia: Acta Médica Colomb [Internet]. 2019 Sept 30;44(4).

• Precision (1.18%) is stated without justification for its selection. Reframe the calculation as an approximate justification, not a strict inferential guarantee.

Thank you for pointing this out. The precision value of 1.18% was not intended as a strict inferential guarantee but rather as an approximate justification of the sample size adequacy (the expected margin of error under the assumptions used). We have revised the text to clarify that this is in lines 177 to 182.

• The logic of applying population-level prevalence to a non-probabilistic hospital-based sample is weak.

We acknowledge the reviewer’s concern. Because the study employed a non-probability hospital-based sampling strategy, the sample size calculation based on population-level prevalence should be interpreted as an approximate guide rather than a direct inferential basis. We have revised the Methods to explicitly state this limitation and to emphasize that the findings may not be fully representative of the broader population. This clarification can be found in lines 183 to 185.

• Cronbach’s alpha of 0.3 for practices is unacceptable, indicating poor internal consistency. Reframe the tool as partially validated.

Thank you for your comment. The term “validated” has been reframed throughout the abstract and main text, and the questionnaire is now properly described based on the above suggestions.

• Despite this, practices are used extensively in regression analyses. Acknowledge that findings related to practices should be interpreted with caution.

Thank you for your comment. We have now acknowledged that the observed poor internal consistency of the practices component of the tool implies that the corresponding results should be interpreted with caution. This clarification has been added to lines 203–206 in the data collection tool section.

• The questionnaire is described as “validated,” which is misleading. Consider removing or downgrading claims based on the practices scale.

Thank you for clarifying this. We have removed the term “validated” throughout the abstract and main text and described the questionnaire accordingly based on the above suggestions.

• The data collection tool mentioned that a systematic review was conducted. Please provide that systematic review in the reference section.

Although a thorough systematic review was conducted, it has not yet been published or submitted for publication. Therefore, we have revised the data collection tool section and reformulated the wording to limit detailed descriptions of this process, given that the review is not currently publicly available. Lines 187-193.

• Please clarify who was responsible for data collection, whether trained research assistants, volunteers, or other personnel.

Thank you for pointing this out. Data collection was conducted by trained nurses, with multiple follow‑ups and audits throughout the process to ensure the reliability of the information obtained. This was now clarified in lines 209-210 in the main manuscript.

Results

• Please provide a table of sociodemographic characteristics of the respondents.

Thank you for your interest in this data. We have now provided a table with sociodemographic characteristics in the supplemental material (table 2), since we have a limited number of tables allowed to share in the main manuscript. This is specified in lines 285-286.

• Minor inconsistencies in denominators across tables. Ensure consistency in denominators.

We thank the reviewer for this observation. We have carefully re-verified all denominators across the tables. We can confirm that they are now correct, specifying the values for every single variable.

• Some percentages are reported without clear numerators.

Thank you for this comment. The total values of responders for each variable were reviewed and specified. The total number of responders was specified as the n value at the end of the statements.

• The Results occasionally interpret findings rather than strictly reporting them. Move interpretative statements to the Discussion.

Thank you for your comment. We carefully revised the Results and Discussion sections to ensure a clear distinction between reporting and interpretation. While our intention in the Results was to describe the observed associations rather than interpret or analyze their implications, we have adjusted the wording throughout these sections to further improve clarity.

Discussion

• Claims of being the “first” in Latin America may be overstated without systematic verification. Moderate novelty claims.

Thank you for this comment. Prior to conducting this study, we performed an extensive literature review and did not identify any published studies addressing stroke knowledge and perceptions among populations with vascular risk factors in Colombia or Latin America. To reflect this more cautiously, we have revised the statement in the manuscript in lines 396 to 399.

• The discussion downplays the serious limitation posed by the low reliability of the practices scale. More explicitly discuss how measurement limitations affect conclusions.

Thank you for pointing this out. Throughout the discussion, modifications were made to better expose the importance of this study without downplaying the limitations linked to the methodology of the type of study and analysis.

• Some arguments drift into health system performance without direct data support. Focus discussion tightly on findings directly supported by data.

Thank you for this valuable comment. Paragraphs between 384 and 395 lines were modified to explain how the results of the study brought these public health matters to the discussion, having in mind the limitations of the results and the main conclusions that could be derived from this.

Conclusions

• Conclusions occasionally imply causality, which is not supported by a cross-sectional design. Rephrase conclusions to emphasize associations rather than causal effects.

Thank you for addressing this point. Conclusions were rephrased to show the importance of the present investigation without compromising causality in it.

Reviewer #2

- The manuscript describes a technically sound observational study, and the data largely support the authors’ conclusions. The study uses an appropriate cross-sectional design to assess knowledge, perceptions, and practices related to stroke among high-risk patients. The large sample size (n=5,402), inclusion of multiple healthcare subnetworks, and use of multivariable logistic regression support the internal validity of the findings. The statistical analyses are appropriate, and the results are clearly presented with consistent reporting of effect estimates and confidence intervals.

The conclusions regarding associations between lower stroke knowledge and male sex, lower education, and lower socioeconomic status are supported by the data. The associations observed between sociodemographic factors and self-reported emergency care practices are also consistent and in line with previous studies. However, some conclusions would benefit from more cautious wording. The cross-sectional design does not allow causal conclusions, and real-world delays in care or clinical outcomes were not directly measured. In addition, the low internal consistency of the practices domain (Cronbach’s alpha = 0.3) limits the strength of conclusions related to behavioral responses. These limitations should be more clearly reflected in the conclusions.

Overall, the study meets the technical standards for publication, provided that the interpretation of findings, especially those related to practices, is appropriately cautious.

- The statistical analysis is generally appropriate, but only partially rigorous. While suitable methods were used, several key variables, such as socioeconomic status, education, and income, were simplified into binary categories for analytical convenience, which may have reduced analytical precision and obscured potential gradients. The authors should explicitly acknowledge that socioeconomic status, education, and income were dichotomized for analytical simplicity, and note that this approach may have reduced analytical precision and obscured potential gradients in stroke knowledge and practices. No additional analyses are required, but interpretations of these findings should be correspondingly cautious.

Thank you for this observation. In response, we have updated the Data Analysis section of the methods, explicitly acknowledging that socio -economic status, income, and education were dichotomized for analytical simplicity, and that this approach may have reduced analytical precision and obscured potential gradients in stroke knowledge and practices. This is found in lines 248 to 256.

Moreover, we rephrased some paragraphs in the discussion and the conclusions to better fit the scope and limitations of the study. This can be seen mainly in 411-424.

- The authors state that all relevant data are included within the manuscript and its Supporting Information. Summary statistics and regression results are clearly reported and sufficient to support the main findings. However, the underlying individual-level data (e.g. anonymized questionnaire responses or datasets used to generate the reported estimates) are not directly provided as supplementary files nor deposited in a public repository. While this may be acceptable given privacy considerations, the data availability could be improved by clarifying whether de-identified raw data can be shared and, if so, under what conditions.

Thank you for pointing this out. In response to this comment, we have clarified out the data availability (lines 440 to 445). We specify that de-identified raw data will be available for qualified researchers under reasonable request, which includes a legitimate academic purpose and agreement to ensure privacy of the data.

- The manuscript is generally well organized, intelligible, and written in clear, standard English. The structure follows a logical flow, and the aims, methods, results, and conclusions are easy to follow. The language is appropriate for an international scientific audience, and the main messages are communicated clearly.

However, minor grammatical issues, occasional awkward phrasing, and some repetitive or overly long sentences are present, particularly in the Discussion section. These issues do not impede understanding but should be addressed during revision.

Thank you for your comment. We have thoroughly revised the discussion section to improve grammar and

structure of the sentences, allowing a clearer understanding of the presented ideas.

Reviewer #3:

It is an interesting study conducted for the first time in Columbia. The study captures the larger number of population showing the representativeness. I learn that this study generates evidence for further programs in this area in Columbia and elsewhere.

Thank you so much for taking the time to review our manuscript. We hope this advances science in stroke and health literacy.

Attachment Submitted filename: Response to Reviewers (1).docx

# Peer review: Decision Letter 1

**DOI:** 10.1371/journal.pone.0335001.r003

**Authors:** Nafisa M. Jadavji

27 Jul 2026

Dear Dr. Oyola,

Thank you for submitting your manuscript to PLOS ONE. After careful consideration, we feel that it has merit but does not fully meet PLOS ONE’s publication criteria as it currently stands. Therefore, we invite you to submit a revised version of the manuscript that addresses the points raised during the review process.

==============================

Please submit your revised manuscript by Sep 10 2026 11:59PM. If you will need more time than this to complete your revisions, please reply to this message or contact the journal office at plosone@plos.org. When you're ready to submit your revision, log on to https://www.editorialmanager.com/pone/ and select the 'Submissions Needing Revision' folder to locate your manuscript file.

- A letter that responds to each point raised by the academic editor and reviewer(s). You should upload this letter as a separate file labeled 'Response to Reviewers'.
- A marked-up copy of your manuscript that highlights changes made to the original version. You should upload this as a separate file labeled 'Revised Manuscript with Track Changes'.
- An unmarked version of your revised paper without tracked changes. You should upload this as a separate file labeled 'Manuscript'.

If you would like to make changes to your financial disclosure, please include your updated statement in your cover letter. Guidelines for resubmitting your figure files are available below the reviewer comments at the end of this letter.

If applicable, we recommend that you deposit your laboratory protocols in protocols.io to enhance the reproducibility of your results. Protocols.io assigns your protocol its own identifier (DOI) so that it can be cited independently in the future. For instructions see: https://journals.plos.org/plosone/s/submission-guidelines#loc-laboratory-protocols. Additionally, PLOS ONE offers an option for publishing peer-reviewed Lab Protocol articles, which describe protocols hosted on protocols.io. Read more information on sharing protocols at https://plos.org/protocols?utm_medium=editorial-email&utm_source=authorletters&utm_campaign=protocols.

As the corresponding author, your ORCID iD is verified in the submission system and will appear in the published article. PLOS supports the use of ORCID, and we encourage all coauthors to register for an ORCID iD and use it as well. Please encourage your coauthors to verify their ORCID iD within the submission system before final acceptance, as unverified ORCID iDs will not appear in the published article. *Only*  the individual author can complete the verification step; PLOS staff *cannot*  verify ORCID iDs on behalf of authors.

We look forward to receiving your revised manuscript.

Kind regards,

Nafisa M. Jadavji, PhD, MSc, BSc

Academic Editor

PLOS One

**Journal Requirements:**

1. If the reviewer comments include a recommendation to cite specific previously published works, please review and evaluate these publications to determine whether they are relevant and should be cited. There is no requirement to cite these works unless the editor has indicated otherwise.

2. Please review your reference list to ensure that it is complete and correct. If you have cited papers that have been retracted, please include the rationale for doing so in the manuscript text, or remove these references and replace them with relevant current references. Any changes to the reference list should be mentioned in the rebuttal letter that accompanies your revised manuscript. If you need to cite a retracted article, indicate the article’s retracted status in the References list and also include a citation and full reference for the retraction notice.

[Note: HTML markup is below. Please do not edit.]

Reviewers' comments:

Reviewer's Responses to Questions

**Comments to the Author**

Reviewer #1: All comments have been addressed

Reviewer #2: (No Response)

**********

2. Is the manuscript technically sound, and do the data support the conclusions?

Reviewer #1: Yes

Reviewer #2: Partly

**********

3. Has the statistical analysis been performed appropriately and rigorously?

Reviewer #1: Yes

Reviewer #2: No

**********

4. Have the authors made all data underlying the findings in their manuscript fully available?

The PLOS Data policy

Reviewer #1: Yes

Reviewer #2: No

**********

5. Is the manuscript presented in an intelligible fashion and written in standard English?

Reviewer #1: Yes

Reviewer #2: No

**********

Reviewer #1: Dear Author,

Thank you for carefully revising your manuscript in accordance with the comments and instructions provided. We appreciate the effort you have invested in addressing the revisions.

I am pleased to inform you that your manuscript is now suitable for acceptance. Congratulations on this achievement, and we wish you every success with the publication of your work.

Reviewer #2: Thank you for the revised manuscript. Several previous comments have been addressed. However, the following issues still require correction before the manuscript can be considered for publication.

1. Numerical inconsistencies and discrepancies between tables and text

Some numerators, denominators, percentages, and totals reported in the tables do not appear to reconcile. In addition, the direction of some sex-specific findings described in the Results does not correspond to the values shown in the tables.

Please recheck all principal results directly against the original dataset and revise the Abstract, Results, tables, figures, and Discussion so that all values and interpretations are fully consistent.

2. Incorrect interpretation of the dichotomized Likert responses

The Methods state that “totally agree” was coded as 1 and all other responses were coded as 0. This means that participants who selected “agree” were grouped together with those selecting neutral or disagreement responses.

However, this combined group is described as participants who “would not call emergency services” or who “disagreed” with calling.

Please either present the original Likert response distribution or describe the comparison accurately as “totally agree” versus “all other responses.” Do not interpret the combined group as refusing or disagreeing to call emergency services.

3. Intended responses should not be described as actual practices

The questionnaire assessed what participants said they would do in a hypothetical stroke situation. It did not measure their actual behavior during a real stroke event.

For example, Table 2 is titled as referring to “practices,” although the study measured intended responses.

Please replace terms such as “practices” and “behavioral responses” with “intended care-seeking responses” or “self-reported intended responses” throughout the manuscript, including table and figure titles.

4. Analytical sample and denominators

Different denominators are used across the manuscript, but the reason is not clearly explained.

Please define the final analytical sample and state whether each analysis used complete-case or available-case data. The number of participants included in each table and regression model should be clearly reported.

5. Conclusions extend beyond the measured outcomes

The manuscript discusses possible problems in emergency medical services, transport, hospital readiness, timely access to care, and treatment delays. These outcomes were not measured.

Please present these issues only as possible topics for future research. The Conclusions should be limited to associations between sociodemographic characteristics, stroke knowledge, and intended care-seeking responses.

6. Data Availability statement

The manuscript states that data are available upon reasonable request, while the submission information indicates that the data are included in the Supporting Information. Please make these statements consistent.

7. The manuscript states that surveys were conducted either by telephone or in person, while the ethics statement indicates that written informed consent was obtained from all participants. Please clarify how written consent was obtained from participants interviewed by telephone and confirm that this consent procedure was approved by the relevant ethics committee.

Overall, I recommend major revision. The principal concerns can be resolved by verifying all reported values against the dataset, correcting discrepancies between tables and text, clarifying statistical models, and limiting the conclusions to outcomes directly measured in the study.

**********

what does this mean?). If published, this will include your full peer review and any attached files.

If you choose “no”, your identity will remain anonymous but your review may still be made public.

**Do you want your identity to be public for this peer review?**  For information about this choice, including consent withdrawal, please see our Privacy Policy

Reviewer #1: **Yes:**  Faizul Akmal Abdul Rahim

Reviewer #2: No

**********

[NOTE: If reviewer comments were submitted as an attachment file, they will be attached to this email and accessible via the submission site. Please log into your account, locate the manuscript record, and check for the action link "View Attachments". If this link does not appear, there are no attachment files.]

To ensure your figures meet our technical requirements, please review our figure guidelines: https://journals.plos.org/plosone/s/figures

You may also use PLOS’s free figure tool, NAAS, to help you prepare publication quality figures: https://journals.plos.org/plosone/s/figures#loc-tools-for-figure-preparation.

NAAS will assess whether your figures meet our technical requirements by comparing each figure against our figure specifications.

# Author comment: Author response to Decision Letter 2

**DOI:** 10.1371/journal.pone.0335001.r004

30 Aug 2026

Edits requested for submission:

If the reviewer comments include a recommendation to cite specific previously published works, please review and evaluate these publications to determine whether they are relevant and should be cited. There is no requirement to cite these works unless the editor has indicated otherwise.

- Thank you; there were no recommendations to cite published works.

___________________________________________________________________________________________________________________________________________________________________

Please review your reference list to ensure that it is complete and correct. If you have cited papers that have been retracted, please include the rationale for doing so in the manuscript text, or remove these references and replace them with relevant current references. Any changes to the reference list should be mentioned in the rebuttal letter that accompanies your revised manuscript. If you need to cite a retracted article, indicate the article’s retracted status in the References list and also include a citation and full reference for the retraction notice.

- Thank you; the reference list was reviewed.

___________________________________________________________________________________________________________________________________________________________________

Reviewer #1

Dear Author,

Thank you for carefully revising your manuscript in accordance with the comments and instructions provided. We appreciate the effort you have invested in addressing the revisions.

I am pleased to inform you that your manuscript is now suitable for acceptance. Congratulations on this achievement, and we wish you every success with the publication of your work.

- Thank you so much for taking the time to review our manuscript. We hope this advances science in stroke and health literacy. We will continue working to improve the manuscript and ensure visibility of our findings.

___________________________________________________________________________________________________________________________________________________________________

Reviewer #2

Thank you for the revised manuscript. Several previous comments have been addressed. However, the following issues still require correction before the manuscript can be considered for publication.

1. Numerical inconsistencies and discrepancies between tables and text

Some numerators, denominators, percentages, and totals reported in the tables do not appear to reconcile. In addition, the direction of some sex-specific findings described in the Results does not correspond to the values shown in the tables.

Please recheck all principal results directly against the original dataset and revise the Abstract, Results, tables, figures, and Discussion so that all values and interpretations are fully consistent.

- We express our gratitude to the reviewer for bringing these critical details to our attention. We have undertaken a complete, systematic audit of our original dataset and carefully rechecked every numerator, denominator, percentage, and summary statistic across the manuscript, including the Abstract, Results, Discussion, tables, and figures, to ensure full reconciliation and accuracy.

Specifically, we have implemented the following revisions:

Hypertension Percentages: We corrected the reported hypertension percentages in lines 292 and 293 of the revised manuscript to align precisely with the raw dataset.

Dyslipidemia Terminology: We updated the label from "Dyslipidemia" to "Any Dyslipidemia" throughout the text (line 295) and tables (Table 1) to clearly communicate that this category encompasses both cholesterol and triglyceride abnormalities.

Missing Data and Totals: We explicitly accounted for non-responses in our item-level breakdowns. Specifically, two participants (one male, one female) declined to answer the items regarding calling the emergency hotline, making a medical appointment, and taking a home remedy. Additionally, three participants (two females, one male) declined to answer the item regarding visiting a pharmacy. The overall totals and denominators across our tables now accurately reflect the inclusion of intersex participants as well as those who selected "do not wish to answer." This information was added in the Table 2 footnote.

Correction of Sex-Specific Findings: We are immensely grateful to the reviewer for pointing out the error in lines 315-316 regarding the direction of the sex-specific association. The text previously stated that women were more likely to activate emergency services; this was an error in drafting. The data demonstrate that men were more likely than women to intend to call emergency services. We have corrected the text accordingly, and we have reviewed our Discussion section (lines 366–380) to accurately reflect and contextualize this corrected direction.

___________________________________________________________________________________________________________________________________________________________________

2. Incorrect interpretation of the dichotomized Likert responses

The Methods state that “totally agree” was coded as 1 and all other responses were coded as 0. This means that participants who selected “agree” were grouped together with those selecting neutral or disagreement responses.

However, this combined group is described as participants who “would not call emergency services” or who “disagreed” with calling.

Please either present the original Likert response distribution or describe the comparison accurately as “totally agree” versus “all other responses.” Do not interpret the combined group as refusing or disagreeing to call emergency services.

- Thank you for bringing this to our attention. In accordance with the recommendation, we have thoroughly revised the text across the manuscript to describe our comparisons accurately through the lens of complete agreement ("totally agree") versus all other response options combined ("agree," "neutral," "disagree," and "totally disagree"). Specifically, we have implemented these adjustments in:

Results Section (Lines 300–332): Re-framed all descriptions of intended care-seeking behaviors to explicitly present comparisons as "totally agree" versus "all other responses," removing any language asserting that the comparison group disagreed with or refused emergency care.

Discussion Section (Lines 337–338, 384): Updated our interpretation of participant responses to accurately reflect non-complete agreement (highlighting potential hesitation in care-seeking intent) rather than outright refusal or disagreement.

___________________________________________________________________________________________________________________________________________________________________

3. Intended responses should not be described as actual practices

The questionnaire assessed what participants said they would do in a hypothetical stroke situation. It did not measure their actual behavior during a real stroke event.

For example, Table 2 is titled as referring to “practices,” although the study measured intended responses.

Please replace terms such as “practices” and “behavioral responses” with “intended care-seeking responses” or “self-reported intended responses” throughout the manuscript, including table and figure titles.

- Thank you for this comment. In accordance with your recommendation, we have systematically replaced terms such as "practices" and "behavioral responses" with "intended care-seeking responses" throughout the manuscript. Specifically, we have updated:

Table and Figure Titles: Revised all titles to accurately specify intended care-seeking responses (including Table 2, Figure 3, and the Figure 3 description). An updated figures document will be uploaded as well.

Results Section (Lines 312–329): Adjusted all narrative descriptions of participant choices to reflect self-reported care-seeking intentions.

Discussion Section (Lines 342, 376, and 384): Refined our interpretations to consistently emphasize that the findings reflect intended, rather than actual, stroke care-seeking behavior.

___________________________________________________________________________________________________________________________________________________________________

4. Analytical sample and denominators

Different denominators are used across the manuscript, but the reason is not clearly explained.

Please define the final analytical sample and state whether each analysis used complete-case or available-case data. The number of participants included in each table and regression model should be clearly reported.

- We appreciate you requesting this important clarification. We have updated the Methods and table footnotes to state that we used an available-case analysis approach and to clarify all denominator variations:

Analytical Approach: Participants were included in each analysis where complete data for the variables of interest were available. Exact values are explicitly reported for every table, figure, and regression model, and the explanation for the missing values was stated in the table’s footnote.

Dyslipidemia Sample (N = 5,398): Differs from the full sample (N = 5,400) because one male participant reported no knowledge of dyslipidemia (n = 2,501) and one female participant declined to answer (n = 2,897).

Table 2 Sex Breakdown (N = 5,398): Sums to 2,502 men and 2,896 women. Two participants reporting other sex identities were retained in the overall total (N = 5,400) but excluded from sex-stratified comparisons due to low frequency (lines 283–284).

We appreciate this feedback, which has enhanced the methodological transparency of our paper.

___________________________________________________________________________________________________________________________________________________________________

5. Conclusions extend beyond the measured outcomes

The manuscript discusses possible problems in emergency medical services, transport, hospital readiness, timely access to care, and treatment delays. These outcomes were not measured.

Please present these issues only as possible topics for future research. The Conclusions should be limited to associations between sociodemographic characteristics, stroke knowledge, and intended care-seeking responses.

- Thank you for this important observation. We agree that our conclusions and discussion must strictly reflect the variables measured in our study.

In accordance with your feedback, we have updated lines 348–349 and line 402 to explicitly state that systemic factors (such as EMS transport dynamics, hospital readiness, and treatment delays) were not directly assessed in this study and represent essential directions for future research. Furthermore, phrasing at the beginning of paragraph 3 (line 350) and line 374 already emphasizes "evidence from other settings," ensuring these contextual links are presented strictly as background context rather than direct findings. Moreover, we eliminated the phrase "timely activation of emergency medical services" from the Conclusions section. The conclusions are now strictly limited to our measured outcomes: the associations between sociodemographic characteristics, stroke knowledge, and self-reported intended care-seeking responses. We appreciate this valuable recommendation, which has strengthened the focus and rigor of our manuscript.

___________________________________________________________________________________________________________________________________________________________________

6. Data Availability statement

The manuscript states that data are available upon reasonable request, while the submission information indicates that the data are included in the Supporting Information. Please make these statements consistent.

- We thank the reviewer for identifying this inconsistency. We have updated the Data Availability statement in the manuscript (line 452) to confirm that the dataset is fully available within the Supporting Information files accompanying the submission.

___________________________________________________________________________________________________________________________________________________________________

7. The manuscript states that surveys were conducted either by telephone or in person, while the ethics statement indicates that written informed consent was obtained from all participants. Please clarify how written consent was obtained from participants interviewed by telephone and confirm that this consent procedure was approved by the relevant ethics committee.

- Thank you for highlighting this discrepancy. The manuscript has been updated to distinguish the consent methods employed. Written informed consent was secured for all face-to-face interviews. For participants surveyed via telephone, verbal informed consent was obtained through a protocol specifically approved by the institutional review boards of UHFSFB and the participating hospitals within the governmental health subnetworks of the North, South, and Center-East (Serial number: SNACEI-132. File 73). These clarifications have been integrated into the Ethical Considerations section of the Methods, lines 272 to 274, to ensure accuracy regarding the approved procedures.

Attachment Submitted filename: Response to Reviewers (2).docx

# Peer review: Decision Letter 2

**DOI:** 10.1371/journal.pone.0335001.r005

**Authors:** Nafisa M. Jadavji

2 Oct 2026

Stroke Knowledge and Intended Care-Seeking Behavior Among Adults With Hypertension and Type 2 Diabetes: A Cross-Sectional Study

PONE-D-25-51084R2

Dear Dr. Oyola,

We’re pleased to inform you that your manuscript has been judged scientifically suitable for publication and will be formally accepted for publication once it meets all outstanding technical requirements.

Within one week, you’ll receive an e-mail detailing the required amendments. When these have been addressed, you’ll receive a formal acceptance letter and your manuscript will be scheduled for publication.

An invoice will be generated when your article is formally accepted. Please note, if your institution has a publishing partnership with PLOS and your article meets the relevant criteria, all or part of your publication costs will be covered. Please make sure your user information is up-to-date by logging into Editorial Manager at Editorial Manager® and clicking the ‘Update My Information' link at the top of the page. For questions related to billing, please contact billing support.

If your institution or institutions have a press office, please notify them about your upcoming paper to help maximize its impact. If they’ll be preparing press materials, please inform our press team as soon as possible -- no later than 48 hours after receiving the formal acceptance. Your manuscript will remain under strict press embargo until 2 pm Eastern Time on the date of publication. For more information, please contact onepress@plos.org.

Kind regards,

Nafisa M. Jadavji, PhD, MSc, BSc

Academic Editor

PLOS One

Additional Editor Comments (optional):

Reviewers' comments:

Reviewer's Responses to Questions

**Comments to the Author**

Reviewer #2: (No Response)

**********

2. Is the manuscript technically sound, and do the data support the conclusions?

Reviewer #2: Partly

**********

3. Has the statistical analysis been performed appropriately and rigorously?

Reviewer #2: Yes

**********

4. Have the authors made all data underlying the findings in their manuscript fully available?

The PLOS Data policy

Reviewer #2: Yes

**********

5. Is the manuscript presented in an intelligible fashion and written in standard English?

Reviewer #2: Yes

**********

Reviewer #2: Thank you for revising the manuscript substantially and addressing most of the substantive concerns raised in the previous round. I consider the study potentially suitable for publication, providing that the following corrections are made:

My main remaining concern is the clarity and consistency of the statistical model specification. The Methods state that the logistic regression models were adjusted for age, sex, and history of hypertension, whereas the legends for Figures 2 and 3 describe adjustment for age and history of hypertension, with sex and other sociodemographic variables presented as explanatory variables. The manuscript should clearly specify the variables included in each multivariable model, particularly for the intended care-seeking outcomes shown in Figure 3. This is necessary to substantiate the use of the term “independently associated.”

I also noted a few remaining numerical inconsistencies that should be corrected before acceptance. For example, the Abstract reports 52% of participants would schedule a medical appointment, whereas the Results and Table 2 report 48.0% (2590/5400). The proportion reporting no knowledge of stroke symptoms is reported as 32.6% in the Results, while a different value appears in the Abstract. These should be reconciled throughout the manuscript.

The interpretation of the findings is substantially improved, particularly the distinction between intended care-seeking responses and actual behaviour. Nevertheless, some statements still go beyond the outcomes directly measured. The study did not measure actual care-seeking behaviour, time to hospital presentation, treatment delay, or reperfusion eligibility. Therefore, statements linking the observed intended responses to actual treatment delays should remain clearly framed as potential implications or hypotheses rather than findings of the present study.

The questionnaire limitation is appropriately acknowledged, including the low internal consistency of the intended care-seeking/practice domain (Cronbach’s α=0.30). Given this limitation and the dichotomization of the Likert responses into “totally agree” versus all other responses, the conclusions regarding intended care-seeking responses should remain appropriately cautious.

I would also ask the authors to resolve the remaining inconsistency in the informed-consent description: the manuscript should clearly distinguish the consent procedure for face-to-face versus telephone interviews.

**********

what does this mean?). If published, this will include your full peer review and any attached files.

If you choose “no”, your identity will remain anonymous but your review may still be made public.

**Do you want your identity to be public for this peer review?**  For information about this choice, including consent withdrawal, please see our Privacy Policy

Reviewer #2: No

**********

# Editor report: Acceptance letter

**DOI:** 10.1371/journal.pone.0335001.r006

**Authors:** Nafisa M. Jadavji

PONE-D-25-51084R2

PLOS One

Dear Dr. Oyola,

I'm pleased to inform you that your manuscript has been deemed suitable for publication in PLOS One. Congratulations! Your manuscript is now being handed over to our production team.

At this stage, our production department will prepare your paper for publication. This includes ensuring the following:

* All references, tables, and figures are properly cited

* All relevant supporting information is included in the manuscript submission,

* There are no issues that prevent the paper from being properly typeset

You will receive further instructions from the production team, including instructions on how to review your proof when it is ready. Please keep in mind that we are working through a large volume of accepted articles, so please give us a few days to review your paper and let you know the next and final steps.

Lastly, if your institution or institutions have a press office, please let them know about your upcoming paper now to help maximize its impact. If they'll be preparing press materials, please inform our press team within the next 48 hours. Your manuscript will remain under strict press embargo until 2 pm Eastern Time on the date of publication. For more information, please contact onepress@plos.org.

You will receive an invoice from PLOS for your publication fee after your manuscript has reached the completed accept phase. If you receive an email requesting payment before acceptance or for any other service, this may be a phishing scheme. Learn how to identify phishing emails and protect your accounts at https://explore.plos.org/phishing.

If we can help with anything else, please email us at customercare@plos.org.

Thank you for submitting your work to PLOS One and supporting open access.

Kind regards,

PLOS One Editorial Office Staff

on behalf of

Dr. Nafisa M. Jadavji

Academic Editor

PLOS One
